# Transposable Element Insertions Are Associated with Batesian Mimicry in the Pantropical Butterfly *Hypolimnas misippus*

**DOI:** 10.1093/molbev/msae041

**Published:** 2024-02-24

**Authors:** Anna Orteu, Marek Kucka, Ian J Gordon, Ivy Ng’iru, Eva S M van der Heijden, Gerard Talavera, Ian A Warren, Steve Collins, Richard H ffrench-Constant, Dino J Martins, Yingguang Frank Chan, Chris D Jiggins, Simon H Martin

**Affiliations:** Department of Zoology, University of Cambridge, Cambridge CB2 3EJ, UK; Tree of Life Programme, Wellcome Sanger Institute, Hinxton, UK; Friedrich Miescher Laboratory of the Max Planck Society, Tübingen, Germany; Centre of Excellence in Biodiversity, University of Rwanda, Huye, Rwanda; Mpala Research Centre, Nanyuki 10400, Laikipia, Kenya; School of Biosciences, Cardiff University, Cardiff CF 10 3AX, UK; UK Centre for Ecology and Hydrology, Wallingford OX10 8BB, UK; Department of Zoology, University of Cambridge, Cambridge CB2 3EJ, UK; Tree of Life Programme, Wellcome Sanger Institute, Hinxton, UK; Institut Botànic de Barcelona (IBB), CSIC-CMCNB, Barcelona, Catalonia, Spain; Department of Zoology, University of Cambridge, Cambridge CB2 3EJ, UK; African Butterfly Research Institute, Nairobi, Kenya; Centre for Ecology and Conservation, University of Exeter in Cornwall, Penryn TR10 9FE, UK; Turkana Basin Institute, Stony Brook University, Stony Brook, NY 11794, USA; Friedrich Miescher Laboratory of the Max Planck Society, Tübingen, Germany; Department of Zoology, University of Cambridge, Cambridge CB2 3EJ, UK; Institute of Evolutionary Biology, University of Edinburgh, Edinburgh, UK

**Keywords:** transposable elements, mimicry, adaptive evolution, linked-reads, bioinformatic methods

## Abstract

*Hypolimnas misippus* is a Batesian mimic of the toxic African Queen butterfly (*Danaus chrysippus*). Female *H. misippus* butterflies use two major wing patterning loci (M and A) to imitate three color morphs of *D. chrysippus* found in different regions of Africa. In this study, we examine the evolution of the M locus and identify it as an example of adaptive atavism. This phenomenon involves a morphological reversion to an ancestral character that results in an adaptive phenotype. We show that *H. misippus* has re-evolved an ancestral wing pattern present in other *Hypolimnas* species, repurposing it for Batesian mimicry of a *D. chrysippus* morph. Using haplotagging, a linked-read sequencing technology, and our new analytical tool, Wrath, we discover two large transposable element insertions located at the M locus and establish that these insertions are present in the dominant allele responsible for producing mimetic phenotype. By conducting a comparative analysis involving additional *Hypolimnas* species, we demonstrate that the dominant allele is derived. This suggests that, in the derived allele, the transposable elements disrupt a cis-regulatory element, leading to the reversion to an ancestral phenotype that is then utilized for Batesian mimicry of a distinct model, a different morph of *D. chrysippus*. Our findings present a compelling instance of convergent evolution and adaptive atavism, in which the same pattern element has independently evolved multiple times in *Hypolimnas* butterflies, repeatedly playing a role in Batesian mimicry of diverse model species.

## Introduction

Butterfly wing patterns are a classic example of adaptive evolution. Evolutionary genetic studies have dissected the loci controlling wing pattern in several species of butterflies from a wide range of ecotypes and families, providing extensive information on the evolution of adaptive traits (Jiggins 2017; Beldade and Brakefield 2018). The genetic architectures uncovered are varied, from supergenes formed by inversions encompassing multiple loci in *Heliconius numata* (Joron et al. 2011) to transposable element (TE) insertions in the peppered moth (van’t Hof et al. 2016) or a gene duplication in the wood tiger moth (Brien et al. 2023).

Key insights on the genetic basis of butterfly wing patters come from the *Heliconius* genus of tropical butterflies (Jiggins 2017). These are best known for the multiple instances of Müllerian mimicry in which several pairs of unpalatable sympatric species converge to the same wing pattern sharing the costs of teaching predators. Numerous studies have looked at the genetic basis of their mimetic patterns, identifying the main genes contributing to these adaptive phenotypes and describing their genetic architecture (Joron et al. 2011; Reed et al. 2011; Martin and Reed 2014; Nadeau et al. 2016; Westerman et al. 2018). Although much is known about the genetic basis of mimicry in *Heliconius*, exploring other systems, particularly those with other evolutionary dynamics such as Batesian mimics, in which palatable mimics resemble toxic models, will provide crucial knowledge on the evolution of adaptive phenotypes.

**Fig. 1.**
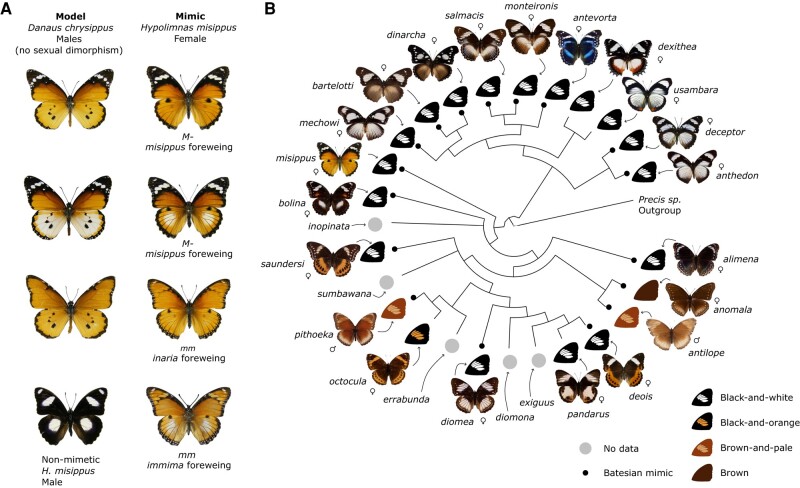
Mimicry in *Hypolimnas missippus* and the *Hypolimnas* genus. A) Female morphs of *H. misippus* side by side with their matching model morphs of *D. chrysippus*. Names of the forewing morph of *H. misippus* are specified below each photo. Although morphs matching the bottom-right *H. misippus* (*immima* forewing and white-spotted hindwing) exist within the *D. chrysippus* hybrid zone, they are considered maladaptive intermediates outside of it. *D. chrysippus* is not sexually dimorphic; individuals shown are all males. Non-mimetic *H. misippus* male at the bottom. B) Phylogram of the *Hypolimnas* genus extracted from Sahoo et al. (2018) (concatenated Bayesian inference tree) showing that black-and-white forewing tips are common through the genus and most likely ancestral. For presentation purposes, one specimen is shown per species, although not all species are monophyletic. Choosing other specimens would not change the conclusion on the ancestrality of black-and-white wing tips. All species shown are sexually dimorphic and/or polymorphic except *antevorta*, *dexithea*, *inopinata*, and *usambara*. Species showing Batesian mimicry are indicated by a small dark dot. Recurrent forewing phenotypes are indicated by wing drawings. Male and female signs indicate the sex of the individual photographed. Butterfly photos are reproduced from Moore (2023) under CC-BY.

The *Hypolimnas* genus of tropical butterflies is diverse in wing pattern phenotypes (Fig. 1B). Interestingly, the genus presents many instances of Batesian mimicry, with the main models being Danaid species of the *Danaus*, *Amauris*, and *Euploea* genera (Vane-Wright et al. 1977). Despite the diversity in phenotype and model species being mimicked, some wing pattern elements are common in most *Hypolimnas*, exemplified by the black-and-white forewing tips found in most species (17/21 species with phenotype data) or the common black or brown background color. These common wing patterns are often adaptive, and it is not known whether they have independently evolved multiple times or are shared through common ancestry. A less likely hypothesis is that these phenotypes are ancestral but have been lost and re-evolved in some species through convergent evolution. This hypothesis is similar to atavism, in which mutations or recombination events recreate an ancestral phenotype using existing developmental machinery, but differs from it in that atavism is often maladaptive. *Hypolimnas* therefore offer an opportunity to study the evolution of adaptive phenotypes in a group that has not been well studied to date.

*Hypolimnas misippus* or Diadem is a pantropical butterfly with complex Batesian mimicry. Females are mimetic and polymorphic, with detailed resemblances to three of the morphs of the toxic African Queen, *Danaus chrysippus* (Fig. 1A; Smith 1973). Despite the striking mimicry, a puzzling mismatch exists in the geographical distribution of *H. misippus* and *D. chrysippus* morphs across Africa, in that the most abundant models are not reflected in the frequency of mimics at a given location (Gordon et al. 2010). This, together with the fact that maladaptive intermediate morphs of *H. misippus* are commonly found, suggests that current selection for mimicry might be weak and raises the question of how the polymorphism is maintained (Gordon and Smith 1998; Gordon et al. 2010). Clarifying the genetic underpinnings of wing mimicry in *H. misippus* will shed light on this complex case of Batesian mimicry and the forces maintaining polymorphism in the population.

Wing coloration in *H. misippus* is determined by two loci of large effect, the M and A loci, determining forewing and hindwing patterns, respectively (Smith and Gordon 1987; Gordon and Smith 1989; VanKuren et al. 2019). The existence of a third locus, the hindwing white suppressor S, has also been hypothesized (Gordon and Smith 1989). The M locus is a Mendelian locus with two alleles, with the dominant M allele (diploid genotype *M*-) producing the mimetic black-and-white forewing tips in the *misippus* morph (Fig. 1A); whereas recessive homozygotes (*mm*) have mimetic orange or intermediate forewings, known as the *inaria* and *immima* morphs, respectively. Epistasis exists between the M and the A loci, producing the intermediate *immima* forms in *mm* genotypes when the dominant *A* allele for white hindwings is present (*mmA*-genotype). Previous work has identified the M locus to an intergenic region of 10 kb near genes of interest such as *pink* and *Sox 5/6* (VanKuren et al. 2019). However, not much is known about the structure of the locus itself, which of the alleles is derived, and whether it arose through de novo mutation or introgression.

Structural variation forms a large part of the genetic variation observed in wild populations and can play a key role in adaptation and speciation (Auton et al. 2015; Wellenreuther et al. 2019). Structural variants (SVs) are typically defined as events larger than 50 bp and include various combinations of gains, losses, and rearrangement of genetic material, which can have extensive effects on gene content, as well as genetic contiguity (reviewed in Ho et al. 2020). These effects have major roles in adaptation and speciation in many species (reviewed in Hoffmann and Rieseberg 2008; Kondrashov 2012; Faria et al. 2019) as well as human disease (Weischenfeldt et al. 2013; Zeevi et al. 2019). For example, inversions have often been associated with complex phenotypes, as reduced recombination at the inversion promotes the joint inheritance of co-adapted alleles (Kirkpatrick and Barton 2006). Examples of this are seen in elytra coloration in ladybirds and reproductive morph switches in the ruff (Küpper et al. 2015; Lamichhaney et al. 2016; Ando et al. 2018; Gautier et al. 2018; reviewed in Thompson and Jiggins 2014; Orteu and Jiggins 2020). In other cases, gene duplications might give rise to adaptive loci through neo-functionalization as seen in heterostyly in *Primula* plants and in the complex phenotypes of the wood tiger moth (Li et al. 2016; Brien et al. 2023).

Despite the importance of SVs in phenotypic variation, their study is limited by the difficulty of detecting them using high throughput “short-read” DNA sequencing (Mahmoud et al. 2019). SVs involve the rearrangement of otherwise identical DNA sequences, so their detection often requires sequencing molecules that span the rearranged sequence junction. Relative to the size of an SV (often >50 kb), the fraction of read molecules (typically 300–500 bp) that span junctions can be vanishingly small. This problem is made worse by ambiguous mapping due to repetitive elements, which contribute to the formations of SVs (Sharp et al. 2005; Carvalho and Lupski 2016; Payer et al. 2017). Nevertheless, a number of programs exist to detect SVs from short-read sequencing (Rausch et al. 2012; Sindi et al. 2012; Layer et al. 2014; Iakovishina et al. 2016). Long-read sequencing, in contrast, has improved our power to detect SVs via reads that span repetitive and problematic regions, but is limited by cost (Sedlazeck et al. 2018; Ho et al. 2020).

Linked-read sequencing has emerged as an alternative that combines the scalability of short-read sequencing while retaining linkage information (Marks et al. 2019). The newly developed “haplotagging” is a simple, linked-read technique that can be used to sequence entire study populations with hundreds of individuals (Meier et al. 2021). In this approach, large DNA molecules are barcoded as they are broken up for short-read sequencing. To detect SVs, the barcoded, larger DNA molecule greatly boosts the fraction of junction-spanning molecules, thus improving detection power. Importantly, haplotagging can be easily scaled up to population level by multiplexing, which makes it possible to track the frequency of polymorphic SVs in single individuals, making it an ideal tool for the study of adaptation and speciation in non-model organisms (Meier et al. 2021).

Here, we dissect the genetic architecture of an adaptive polymorphism in *H. misippus* using haplotagging data. First, we describe our custom program, WRapped Analysis of Tagged Haplotypes (Wrath, github.com/annaorteu/wrath), and validate in two ways: (1) we run Wrath on published *Heliconius* haplotagging data with known SVs and (2) we test it against simulated *Heliconius* data. Thereafter, we focus on the *H. misippus* case by first performing an association study using hundreds of whole genome haplotagging sequences to pinpoint the candidate locus controlling mimicry in this system. We then use the linked-read information to dissect the genetic structure of the locus by applying Wrath. Finally, we perform a cross-species comparison within the genus *Hypolimnas* to investigate the evolutionary history of the wing pattern mimicry alleles.

## Results

### Wrath: A Tool for Visualizing and Detecting Candidate SVs From Linked-Read Data

We describe Wrath, our program for the visualization and exploration of SVs from linked-read data. Wrath divides chromosomes into genomic windows and quantifies barcode sharing among them, creates a matrix and heatmap that can be used to identify outlier regions caused by SVs, and performs statistical testing to identify candidate SVs (see Methods for a more detailed description). We validate Wrath’s accuracy with two approaches: (1) we apply Wrath to an existing haplotagging dataset of *Heliconius* butterflies (Meier et al. 2021) and (2) we create a simulated dataset of known SVs by inducing SVs of a range of sizes (1, 5, 10, 50, and 100 kb) on the *Heliconius melpomene* genome (v2.5) and test the sensitivity of Wrath at detecting them using the Meier et al. (2021) dataset.

**Fig. 2.**
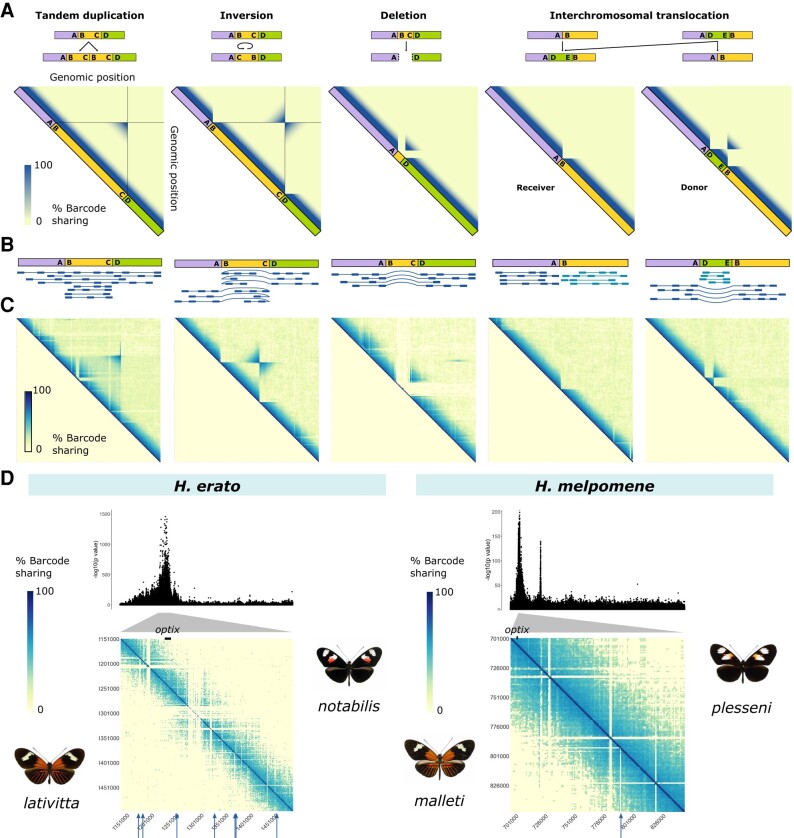
Wrath produces visualizations of linked-read data to identify candidate SVs. A) Hypothetical Wrath outputs for some SV types showing heatmaps of barcode sharing between genomic windows of a chromosome. On top of each heatmap are depicted the reference genome and its order of loci, and below a depiction of the rearranged genome region containing an SV. Points A, B, C, and D depict different loci around the breakpoints of the SV that we use as a guide through the diagrams. B) Linked-read mapping pattern on the reference genome for each of the hypothetical SVs. C) Wrath output heatmaps from the *Heliconius* dataset depicting possible SVs that match the hypothetical predictions. Plots are produced using all *H. melpomene* (Hmel) or *H. erato* (Herato) at a time, focusing on the following genomic positions (left to right): Hmel208001o:6150000-6850000, Hmel213001o:4850000-5550000, Herato2101:12790000-13490000, Hmel214004o:2750000-3450000, and Hmel220003o:10150000-10850000. D) Wrath outputs of the region around one of the loci (the *optix* locus) associated with color pattern in *H. erato* (left) and *H. melpomene* (right). Each triangle half of the matrix depicts barcode sharing for one of the color pattern subspecies—in each case depicted by the side. Arrows point at putative deletions that are polymorphic between the two subspecies. Above the heatmap is plotted the Manhattan plot of the result of the Genome Wide Association Study (GWAS) of color pattern between each pair of subspecies. These show only the region around the associated *optix* locus (Herato1801:1000000-1500000 for *H. erato* and Hmel218003o:700000-850000 for *H. melpomene*). Triangles depict the correspondence of regions between the GWAS and heatmap. Black rectangles depict the location of *optix*.

To validate Wrath, we first use it on an available dataset of the two tropical butterfly species *Heliconius erato* and *H. melpomene* (Meier et al. 2021). Each species presents two morphs or subspecies with mimetic wing patterns which hybridize: *H. melpomene plesseni* and *Heliconius malleti*, and *H. erato notabilis* and *Heliconius lativitta*. We used this dataset to explore which SVs are found in these populations. First, we searched for any SVs present in the dataset genome wide and identified 2,284 large (>10 kb) putative SVs in *H. melpomene* and 2,265 in *H. erato* (Supplementary Material online). We then explored these using the heatmaps produced by Wrath. Patterns of barcode sharing observed in the heatmaps can be used to identify the type of SV present in the samples (Fig. 2A–C). For example, inversions result in a bowtie pattern in the heatmap, as more barcodes are shared than expected between loci that are far apart in the reference genome. We produced heatmap plots for all large scaffolds of *H. melpomene* and *H. erato* and explored the SVs present in the dataset (Fig. 2C and Supplementary Material online). For example, a known SV in chromosome 2 in *H. erato* (Meier et al. 2021) was clearly visible in the heatmap (supplementary fig. S1, Supplementary Material online). With this, we show that Wrath can visualize patterns of barcode sharing and help prioritize the order of exploration of SVs, as visual examination of the haplotagging data helps explore the SV content in the samples. Nonetheless, some SVs produce similar or matching signals. For example, patterns of interchromosomal translocations like that shown in Fig. 2A can also be produced by TE insertions.

Finally, we assessed the time required to run on the dataset. In a small subset of the data and running with 20 threads, the parallel implementation ran 10× faster than a single-threaded implementation of the same algorithm (supplementary fig. S2, Supplementary Material online).

### Wrath Identifies SVs From Simulated Data, But Is Limited by Molecule Size and Window Size

To further test Wrath’s accuracy, we applied it to simulated data with known SVs. To do that we induced 710 SVs of a range of sizes (1, 5, 10, 50, and 100 kb) on the *H. melpomene* genome. We then mapped the *H. melpomene sp. plesseni* samples from Meier et al. (2021) and tested the sensitivity (true positives/(true positives + false negatives)) of Wrath at detecting them using two genomic window sizes, 10 and 5 kb. First, we defined a measure of error to categorize the SVs identified by Wrath as correct or incorrect. To do that we measure the absolute distance between detected and simulated breakpoints and scale it by genomic window size to make the measure easier to interpret (supplementary fig. S3, Supplementary Material online). We use this scaled error measure to evaluate the sensitivity of Wrath. SVs equal or larger than 50 kb were detected with low errors and high sensitivity using either window size (for threshold = 20 windows, sensitivity ≥0.981, mean = 0.994 for 10 kb and ≥0.846, mean = 0.877 for 5 kb windows; supplementary figs. S4 and S5, Supplementary Material online). On the other hand, Wrath largely failed at recovering SVs of 10 kb or smaller size. There are two reasons for this: window size and molecule size. Smaller genomic window sizes allow for the detection of smaller SVs, and their breakpoints are detected with higher precision (i.e. distance between the identified breakpoint and the real one is smaller). Using a 5 kb window, we were able to identify most simulated SVs of 50 kb size or larger within 20 windows. However, smaller genomic windows come at a computational cost and produce noisier results, that is, if coverage is not high enough, there might not be enough barcodes in each window to pick up signals of SVs with barcode sharing. A second limitation to the detectability of SVs particularly for inversions is molecule size, which in this dataset ranges from 40 to 60 kb (Meier et al. 2021). SVs that are much smaller than the molecule size (e.g. 10 kb) will not have any difference in barcode sharing at either side of the breakpoints, which is the signal used to detect certain rearrangements such as inversions. Thus, molecule size poses a lower limit on the size of certain type of SVs that can be detected using linked-read data. Finally, for very large inversions (i.e. 100 kb), sensitivity of the detection is correlated with window size, because smaller windows identify the edges of the bowtie pattern, which are not equivalent to the breakpoints. However, the signal of depletion of barcode sharing that the inversion breakpoint has at the diagonal (second case in Fig. 2A–C) will be identified as a separate SV by Wrath, which can be used to accurately identify the breakpoint.

### Multiple Deletions Are Found at the *Optix* Color Locus *H. melpomene* and *H. erato*

Following the genome wide analyses of the *Heliconius* dataset, we were interested in visualizing barcode sharing and candidate SVs at a local scale, particularly to explore whether known wing patterning loci in *Heliconius* were also associated with structural variation. Using Wrath, we found that in both species pairs, there are multiple putative deletions of 1–10 kb that differentiate color morphs at the locus associated with red pattern elements near the gene *optix* (Fig. 2D). Deletions leave an area depleted of barcode sharing, as reads do not map to the area, which can be visually identified using the heatmaps (Fig. 2A–C). However, only polymorphic deletions can be identified, as deletions that are fixed in all sequenced individuals but not in the reference cannot be distinguished from assembly artifacts or poor mapping (e.g. repetitive regions for which mapping reads are filtered out due to low mapping quality). Larger deletions (>10 kb) leave an additional signature in the heatmap, showing increased barcode sharing between the breakpoints, in the shape of a triangle (Fig. 2A–C). This is because more molecules than expected span through the breakpoints in individuals presenting the deletion. This signature is harder to detect in smaller deletions, as barcode sharing across the small, deleted region is similarly high in individuals with and without the deletion.

The red patterning locus contains cis-regulatory elements that control the expression of *optix* and influence development of red color elements, which have been functionally tested with CRISPR (supplementary fig. S6, Supplementary Material online; Lewis et al. 2019). Thus, one possibility is that these deletions are disrupting the function of an *optix* cis-regulatory element (CRE) and affecting *optix* expression, although they will need to be functionally tested. Alternatively, it could be that these deletions are in linkage with selected single nucleotide polymorphisms (SNPs) and thus associated with the adaptive color patterns. Finally, the deletion detected in *H. melpomene* was detected previously using short-read sequencing of two different subspecies (Wallbank et al. 2016).

### Forewing Mimicry in *H. misippus* Is Controlled by the M Locus

To explore the genetic underpinnings of Batesian mimicry in *H. misippus*, we first confirmed the previously described identity of the M locus (VanKuren et al. 2019). We sequenced 332 *H. misippus* females collected in distinct locations across Africa (supplementary table S1, Supplementary Material online) using haplotagging. The dataset contains 275 *misippus* (*M-*) individuals and 57 *inaria/immima* (*mm*, supplementary table S2, Supplementary Material online), sequenced to 0.81 coverage on average (supplementary table S3 and fig. S7, Supplementary Material online). By using haplotagging with a large dataset, we could sequence at low coverage per individual without compromising on statistical power to detect loci associated with mimicry. This is because, although read coverage is low, molecular coverage (i.e. coverage of DNA molecules) is higher in linked-read data, as SNP information of reads belonging to the same DNA molecule can be used for imputation and phasing (Marks et al. 2019; Meier et al. 2021). Also, using a large population sample (>200 individuals) facilitates the identification of regions associated with the trait of interest (Lou et al. 2021).

**Fig. 3.**
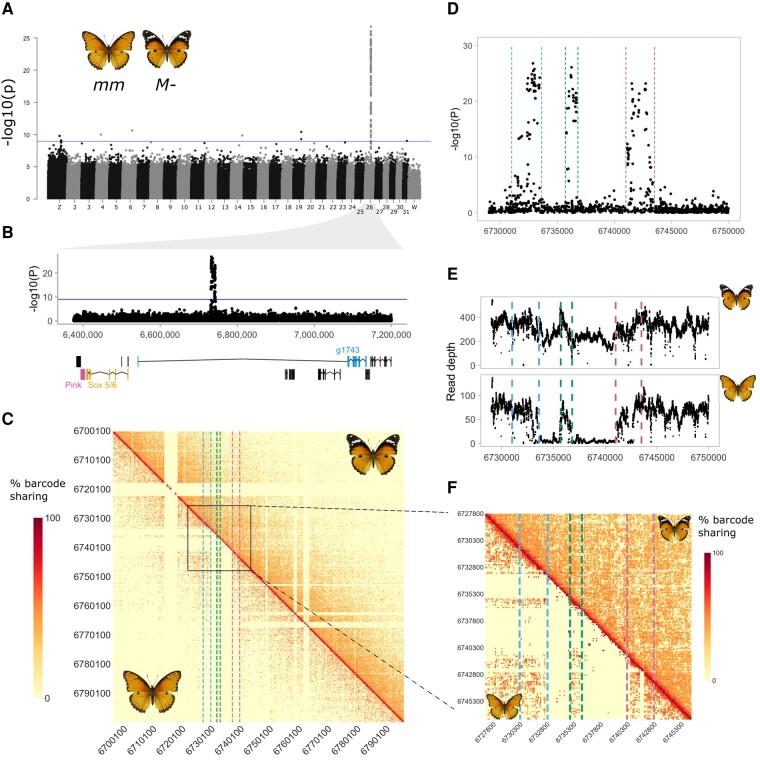
Two large indels are found at the locus associated with forewing mimicry in *mm* individuals. A) GWAS of forewing phenotype shows a unique peak at chromosome 26. Blue line indicates the genome wide significance threshold with Bonferroni correction for multiple testing (*P* = 0.05, *N* = 46,088,305). B) The GWAS peak showing local annotation track with the three main candidate genes colored: *Pink*, *Sox 5/6* and g1743. C) Heatmap of barcode sharing of the region around the association peak. Upper triangle shows barcode sharing for *M-* individuals and lower triangle *mm* individuals. A different pattern of barcode sharing between *mm* and *M-* individuals is seen at the associated region. D) At a finer scale, the association peak reveals a three-peak structure. E) Depth of read coverage around the associated region supports the hypothesis of deletions, as *mm* individuals present almost 0 coverage between the association peaks, while *M-* individuals have more constant coverage throughout the region (note that some *M-* individuals are likely to be heterozygous, carrying one copy of the recessive *m* allele, explaining the partial reduction in read depth in *M-* individuals). F) A zoom in of the barcode sharing heatmap reveals a signal of depletion between the peaks of association in *mm* individuals, a signature of deletions relative to the reference (or insertions in the reference).

First, we parsed and demultiplexed the data and then imputed SNPs and phased haplotypes, which resulted in the identification of 46.1 M SNPs, with a mean phased block N50 of 109.02 kbp (supplementary fig. S8, Supplementary Material online). We then performed a Genome Wide Association Study (GWAS) to identify the locus controlling forewing mimicry. A single large peak of association with variation in forewing phenotype was found on chromosome 26 (6,731,000–6,743,400 bp; $\chi^{2}$ [1, *N* = 331] = 118; *P*-value = 1.703 × 10^−27^ of top associated SNP; Fig. 3A), corresponding to the M locus (VanKuren et al. 2019). Principal component analysis (PCA) of the whole of chromosome 26 showed no evidence for population structure in the data (supplementary fig. S9, Supplementary Material online). In contrast, when using just the associated region for PCA, individuals of the same phenotype were found closer together (supplementary fig. S10, Supplementary Material online). Closer examination of the associated region in the GWAS result revealed three separate peaks of association (Fig. 3D), suggestive of a linked haplotype block. By contrast, the intervening SNPs with little to no association occur within tracts with extremely low read depth in individuals with the *inaria* (*mm*) phenotype (Fig. 3E), indicative of an absence of sequence reads matching these tracts in *inaria* individuals. One possibility is that structural variation is segregating at this locus.

### *misippus* Individuals Carry Multiple TE Insertions at the M Locus

To explore structural variation more closely, we applied Wrath to *H. misippus* population data. The region associated with differences in forewing pattern spans 10 kb in length, thus a very small window size is necessary to elucidate whether there is any structural variation at the locus, given that SVs can only be detected if they are smaller than the genomic windows used (Methods). We visualized barcode sharing using a window size of 100 bp around the M locus and identified two putative deletions in the recessive *m* allele relative to the reference genome, which is a haploid assembly generated from an *M* homozygote individual (Fig. 3C and F; see Methods). These two indels perfectly match the locations of the troughs of association seen in the GWAS analysis where read coverage is almost zero in *mm* individuals, supporting the hypothesis of two deletions in the *m* allele (or two insertions in the *M* allele; Fig. 3E). This explains the decline in association in these two regions, as SNPs cannot be confidently called in *mm* individuals.

**Fig. 4.**
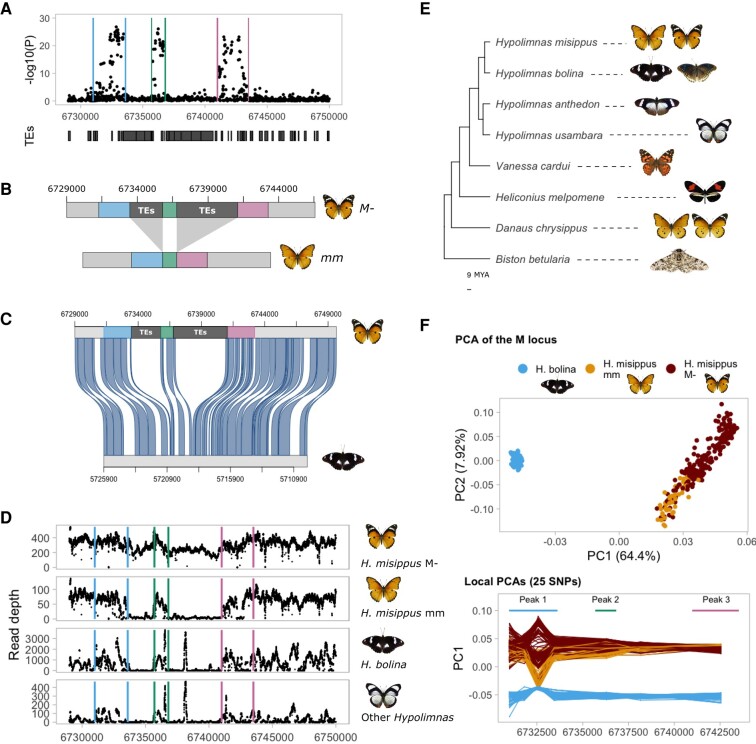
TE insertions are found in the dominant derived allele *m.* A) Zoom in of the association peak showing a TE annotation track. The regions between the association peaks are composed by TE insertions. B) Schematic of the structure of the dominant derived (top) and recessive ancestral M locus alleles. C) The alignment of the reference genomes of *H. misipppus* and *H. bolina* (in reverse orientation) shows that *H. bolina* does not present the TE insertions. D) Read coverage of *H. bolina* and other *Hypolimnas* species suggests that other *Hypolimnas* do not carry the insertions and that those are thus derived. E) A phylogeny of the *Hypolimnas* species used and other Lepidoptera. *H. deceptor*, which is missing, is a sister species to *H. anthedon* (Fig. 1B). Phylogeny extracted from Kumar et al. (2022). F) PCA of the locus associated with forewing phenotype reveals the structure by species, while local PCA in sliding windows of 25 SNPs reveals that the *m* allele is more similar to the *H. bolina* samples in the region of the first peak (chromosome 26: 6732649-6732923), supporting the hypothesis of the recessive allele being ancestral.

To verify the presence of these indels, we designed PCR primers flanking each indel, and at the breakpoints, and amplified them from *misippus* (*M*-) and *inaria/immima* (*mm*) individuals (four individuals per phenotype; supplementary fig. S11, Supplementary Material online). This confirmed that two insertions of 2.4 and 4.3 kb are present in the dominant *misippus* phenotype relative to the recessive *inaria/immima*. A set of TEs insertions detected with RepeatMasker compose the entirety of the two insertions, which are situated in a 3′-UTR intron of the gene g1743, an ankyrin repeat and sterile alpha motif domain containing gene of unknown function (Fig. 3B and A and supplementary tables S4 and S5, Supplementary Material online). Insertion A (most downstream) is composed of a tandem duplication of *Helitron* family TE and an unknown TE, while insertion B is composed of three *Helitrons*, four LINEs, and two unknown TEs (Fig. 4A and supplementary table S6, Supplementary Material online). Given that the insertions are found in the dominant allele, the most plausible explanation is that the insertion is modifying the expression of a nearby gene, either g1743 or others such as *Sox 5/6* and *pink*, by acting as or affecting existing cis-regulatory elements.

### The *M* Allele Carrying TE Insertions Is Derived and Produces an Atavistic Adaptive Phenotype

We next explored the evolutionary history of the M locus. The presence of the TE insertions could either be ancestral or derived. In other words, either the ancestor of both alleles already carried these TE insertions, and they were subsequently deleted to form the *m* allele as it is today, or the ancestor lacked the TE insertions, and they were inserted to form the *M* allele as it is today. To test these hypotheses, we explored this region in the genome of *Hypolimnas bolina*, a relative of *H. misippus* with very different wing patterns that diverged approximately 8 million years ago (Sahoo et al. 2018). First, we aligned the *H. misippus* and *H. bolina* reference genomes using Satsuma2, an aligner intended for inferring homology from sequence similarity (Grabherr et al. 2010). We identified an orthologous region on chromosome 26 of *H. bolina* showing strong synteny across the M locus, with no further rearrangements except for the TE insertions, indicating conserved synteny in *H. misippus* (Fig. 4C). Furthermore, the alignment shows that, while the peaks of association have homologous sequences in chromosome 26 in *H. bolina*, the two indels between the peaks have no matches in the *H. bolina* genome (Fig. 4C). This strongly suggests that the *m* allele is ancestral, and the TEs represent derived insertions into the *M* allele.

To further explore the origin of the alleles, we analyzed whole genome resequencing data from 4 other *Hypolimnas* species, including 214 *H. bolina*, 4 *Hypolimnas anthedon*, 4 *Hypolimnas deceptor*, and 2 *Hypolimnas usambara* (see Fig. 4E for a phylogeny), sequenced to an average coverage of ∼6.5×. First, we mapped all resequenced *Hypolimnas* to the *H. misippus* reference genome and quantified read coverage at the M locus. Read coverage across the TE insertions is approximately zero in the outgroup species (Fig. 4D), confirming the hypothesis that the TE insertions are derived and unique to the *M* allele of *H. missippus*.

To investigate in more detail the evolution of the *M* and *m* alleles, we evaluated the SNP variation at the locus across all the *H. misippus* and *H. bolina* samples using PCA. PCA was conducted using the SNPs of the associated region, and then repeated using windows of 25 SNPs across the region. We chose to use PCA because compared to other techniques such as phylogenetic trees, PCA or similar dimension reduction techniques focus on the main mode of variation and can be more robust under low-coverage sequencing scenarios such as our dataset. Here, our aim is to identify overall genetic similarity: in broader regions, a PCA should recapitulate the species relationships and separate all *H. misippus* from all *H. bolina* samples. However, if the derived *M* allele accumulated an excess of derived mutations, we may find certain tracts in which the *inaria/immima* individuals, which carry the ancestral *m* allele, group more closely with the *H. bolina* individuals than with the *H. missipus* individuals carrying the *M allele*.

Across the whole associated region that contains the M locus, the PCA reflects the species relationships, with one cluster for each species (Fig. 4F, top). Contrastingly, performing local PCAs in genomic windows of 25 SNPs reveals a different pattern. We identified a region containing the top associated SNPs of the most upstream peak of association (6,732,649–6,732,923) for which the relationship between the samples did not reflect the species relationship (Fig. 4F, bottom). *Inaria/immima mm* individuals are found closer to *H. bolina* than to *misippus M*- individuals, which could suggest that the *m* allele is ancestral. The TE insertions found in the *M* allele have reduced recombination, because of reduced effective population size, which could lead to the reduction of recombination at the flanking regions. The TEs might be disrupting a functional element and thus under weak selection which could be coupled with the low recombination and lead to the accumulation of mutations. These coupled effects could make the *M* allele retain fewer ancestral SNPs at the flanking region than the *m* allele. Taken all together, the read coverage, reference genome alignment, and local PCA at the associated region suggest that the recessive *m* allele that produces orange forewings in homozygosis is ancestral to the dominant *M* allele that produces black-and-white forewing tips.

## Discussion

Here, we present a case of adaptive atavism in the diadem butterfly, *H. misippus*, in which the derived allele is associated with a reversion to an ancestral yet adaptive phenotype. Atavisms are caused by mutational or recombination events that enable the pre-existing developmental machinery to reproduce the ancestral character (Hall 2010). Crucially, they are often maladaptive, as the lost phenotype has been selected against, such as hind limbs in whales and teeth in birds, or are associated with a malfunctional state such as cancer (Thomas et al. 2017). In line with this, Stephen Jay Gould revisited Dollo's law, which refers to the paleontological observation that morphological traits that are lost in an evolutionary lineage do not later on re-evolve in that lineage (Gould 1970). We present a case where the atavistic phenotype is adaptive, with the derived allele of the M locus in *H. misippus* producing a mimetic wing phenotype. We show that two large insertions of 2.4 and 4.3 kb are found in the dominant allele of the M locus and that these are formed by multiple TE insertions. By comparison to other *Hypolimnas* species, we show that the insertions are derived. Our results suggest that, from an ancestral black-and-white forewing morph, an orange morph evolved in *H. misippus* by a mutation in an unknown locus, and that this morph reached fixation in the population. Following that, TE insertions at the M locus created the *M* allele, which reverted the phenotype to the ancestral black-and-white forewing morph. Melanised apexes in the forewing with subapical white bands (i.e. forewings with black-and-white tips) are a common wing phenotype in *Hypolimnas* present in 81% of the species (17 out of 21 with phenotype data; Fig. 1B) and in Nymphalids such as Danaids or some *Nymphalinae*, including *Antanartia* and *Vanessa species* (e.g. *Vanessa cardui*; Fig. 4D). Here, we can understand the phenomenon of “evolutionary reversion” in the *H. misippus* butterfly as a molecular example of convergent evolution that is the re-evolution of the same (ancestral) phenotype via regulatory rewiring. Under this model, the original mutation that caused the change to orange wings would not be identifiable by sampling wild *H. misippus*, as this mutation fixed deep in the past, prior to the emergence of the *M* allele.

In summary, we show that *H. misippus* is an example of adaptive atavism in which the TE insertions in the derived *M* allele cause a reversal to an ancestral phenotype, the black-and-white phenotype, which has an adaptive function in Batesian mimicry. Adaptive atavism is a rare event with only a few known examples such as the re-evolution of wings in stick insects (Whiting et al. 2003) and aphids (Saleh Ziabari et al. 2023), sexual reproduction in oribatid mites (Domes et al. 2007), and shell coiling in gastropods (Collin and Cipriani 2003). Alternatively, it could be that the TE insertions are not directly causal but in linkage disequilibrium with the causal mutation. Functional testing such as gene editing with CRISPR-Cas9 would be necessary to prove the causality of the TE insertions. Furthermore, introgression of the *m* allele from another *Hypolimnas* species such as *H. bolina* could explain the local PCA results. However, phylogenetic trees of those SNPs do not show any introgression signal, that is clustering of *H. bolina* samples with *H. misippus m* alleles (Supplementary Material online).

The TE insertions identified are found in the intron of the gene g1743, which encodes an ankyrin repeat and sterile alpha motif domain containing protein of unknown function. Our results represent a similar case to the peppered moth, where a 22-kb TE insertion increases the expression of *cortex*, resulting in the production of melanic morphs (van’t Hof et al. 2016). Similarly, variation in wing color in the *H. melpomene/timareta* lineage is associated with a TE insertion in the *cis*-regulatory region of *cortex*, suggesting that cis-regulatory structural variation controls these mimetic phenotypes (Livraghi et al. 2021). Outside of Lepidoptera, TE insertions in cis-regulatory regions have also been found to be of adaptive importance, such as in egg-spot phenotypes in cichlid fish (Santos et al. 2014) and flowering time in the annual and inbreeding forb *Capsella rubella* (Niu et al. 2019). Given that the M locus insertion is found in a non-coding region, the most plausible explanation is that the insertion is modifying the expression of a nearby gene, either g1743 or others such as *Sox 5/6* and *pink* by acting as or affecting existing cis-regulatory elements. Also, given that the insertion is found in the dominant allele, an increase in expression of the candidate gene or a certain isoform are possible explanations, which could be achieved by the disruption of a repressor or the generation of a novel enhancing function. Overall, this case adds more evidence that cis-regulatory mutations are associated with pattern variation, while coding mutations are more likely to be associated with color, and sheds light on the adaptive importance of TEs (Casacuberta and González 2013; van’t Hof et al. 2016; Orteu and Jiggins 2020).

Although the accuracy of the mimicry seen in *H. misippus* would suggest strong selection, intermediates are often found (Gordon et al. 2010). Moreover, while the subspecies of the model *D. chrysippus* each have large geographically distinct regions in which they are monomorphic (Liu et al. 2022), the three *H. misippus* mimetic morphs are found in all these regions. In other words, there is a phenotypic mismatch, in which many *H. misippus* individuals are mimics of a model that is rare or absent in their region, suggesting that other evolutionary forces might be at play (Gordon et al. 2010). Negative-frequency dependent selection has been invoked as one of several forces maintaining the polymorphism in *H. misippus* (Gordon 1987; Gordon et al. 2010). Another alternative is that structural variation causes associative overdominance and prevents the fixation of a single allele even in scenarios of weak mimicry selection. An example of this has been shown in *H. numata* in which a supergene containing three inversions controls wing phenotype (Jay et al. 2021). The inversions result in a region of low recombination, which in turn lead to the accumulation of deleterious recessive mutations. This leads to reduced fitness of homozygotes for both inversion alleles due to the deleterious effects of recessive mutations at different, but tightly linked sites (i.e. associative overdominance). The TE insertions at the *M* allele of *H. misippus* could theoretically lead to associative overdominance by reducing local recombination between *M* and *m* alleles, but the impact on recombination is likely to be less severe than that of a large inversion, so the role of recombination suppression on the maintenance of polymorphism requires further investigation.

In addition to our empirical results, we also present Wrath, a user-friendly, flexible, and fast tool for the visualization of haplotagging data and exploration of candidate SVs, which we test using two large haplotagging datasets and simulated data. Wrath produces heatmap plots of barcode sharing that can be used to visually inspect the data and identify candidate SVs. Wrath can be run with any chosen window size, which gives flexibility to the user and allows for the detection of SVs of different sizes. There are multiple software solutions for the identification of SVs from linked-read data, including LongRanger (Sudmant et al. 2015), Leviathan (Morisse et al. 2021), NAIBR (Elyanow et al. 2018), and GROC-SV (Spies et al. 2017). LongRanger and GROC-SV are very well curated tools for the analysis of linked-reads produced by 10×-Genomics, while NAIBR uses the BAM (Binary Alignment Map) files produced by LongRanger for its SV detection pipeline. Whilst these programs could be used for haplotagging data, the data would need to be converted to the 10×-Genomics format for input to these programs. Finally, Leviathan can take haplotagging data as input and produces a list of detected candidate SVs and their predicted breakpoints; however, unlike the above tools, Leviathan does not produce graphic visualizations of barcode sharing, which we have found a very useful tool for manual verification of putative SVs. We tested the effectiveness of Wrath using our dataset of *H. misippus*, a published dataset from *Heliconius* butterflies and simulated data, and show that Wrath can be useful in the visualization of haplotagging data to identify candidate SVs.

Altogether, our study presents a striking case of adaptive atavism in which an ancestral trait reappears for an adaptive function. Furthermore, our results highlight the importance of structural variation in the evolution of adaptive phenotypes, adding to the mounting evidence that TEs have an important role in adaptive evolution and particularly in the evolution of color and mimetic phenotypes in Lepidoptera. Finally, we have shown that Wrath is an easy and flexible means to visualize haplotagging data and explore candidate SVs.

## Methods

### Visualization and Exploration of SVs From Haplotagging Data Using Wrath

To analyze haplotagging data, we developed Wrath (WRapped Analysis of Tagged Haplotypes, available at github.com/annaorteu/wrath), a program for the exploration and visualization of SVs consisting of three steps.

### Barcode Parsing

Haplotagging reads are produced using magnetic beads that present a modified Tn5 enzyme on their surface carrying sequencing adapters, each with a unique barcode. During library preparation, DNA molecules wrap around the beads and are cut into smaller fragments and barcodes attached to them. Thus, reads belonging to the same DNA molecule present the same unique barcode, and the small size of the beads ensures that each barcode combination is unique to one or a small number of molecules. In the sequencing files, barcode information is included as four nucleotide sequences of 6 bp each (two per index read). To analyze haplotagging data, first, molecule information needs to be included as a BX tag in the information fields of the fastq files, a process known as molecule demultiplexing. Once the reads include information on their molecule-of-origin in their BX tag, they are ready to be mapped.

### SV Visualization

Using mapped reads and a reference genome, Wrath plots heatmaps of barcode sharing with a single command. Wrath can also produce a list of candidate SVs. Haplotagging reads belonging to the same DNA molecule present the same unique barcode and are expected to map in close proximity in the genome in the absence of rearrangements. Thus, we can use patterns of barcode sharing between more distant genomic windows that exceed the background expectation to identify SVs.

Wrath divides a given chromosome into *n* windows of size *m* (m needs to be specified, by default 10 kb) and identifies the barcodes attached to the reads mapping in each of the genomic windows. Window size is chosen based on two factors. First, computational overhead, as Wrath builds a matrix of *nxn* dimensions which can require a large amount of memory for large values of *n*. And second, molecule size, which depends on several factors such as sample preservation, DNA extraction, and library preparation. By default, molecules are assumed to be centered around 50 kb in length, although they can be much larger (Meier et al. 2021). Window size needs to be smaller than molecule size (e.g. 10 kpb window size and 50 kb molecule size) as the identification of SVs is only possible if molecules span more than one window.

Once the chromosome has been split into windows, Wrath determines the barcodes that are present in each of those windows and calculates the Jaccard index for each pair of windows along the chromosome and stores the value in a matrix of *nxn* dimensions. The Jaccard index is an index of similarity that quantifies barcode sharing between windows:


$$J(A,B)=\frac{\left|A\cap B\right|}{\left|A\cup B\right|},$$

where *J* is the Jaccard value between window *A* and *B* from a given chromosome.

The highest values are expected around the diagonal, which then decay exponentially with distance from it. This is because windows that are closer to each other are expected to share more barcodes than windows that are further apart, as DNA molecules span more than one window. SVs such as moderate to large inversions (>50 kb), intrachromosomal translocations, and long duplicated regions are expected to deviate from the background distribution of barcode sharing. For example, inversions show up as bowtie patterns of excessive barcode sharing (Fig. 2). We define excessive sharing as barcode sharing that is statistically higher than that expected by the distance between the windows given that barcode sharing decays exponentially from the diagonal. Conversely, SV of a much smaller size than the average molecule length cannot be detected with linked-reads. In those cases, short-read methods are more appropriate.

Finally, the construction of the matrix and calculation of Jaccard indices for each genomic window can be a computationally expensive task, for that Wrath can be run in parallel, which reduces computational time.

### Exploration of Candidate SVs

Wrath has the additional functionality of producing a list of candidate SVs. Wrath detects SVs from the matrix by finding window comparisons with excess of barcode sharing, such as in inversions, interchromosomal translocations, and duplications. In a chromosome without any structural variation compared to the reference genome, windows in close proximity are expected to share more barcodes than windows that are far apart. Thus, in the matrix of barcode sharing, values are expected to be the highest at the diagonal and to decay exponentially with distance from it, following a double exponential decay model. Knowing the distance between each entry and the diagonal and the value of barcode sharing, we can identify outliers, that is candidate SVs. Wrath fits double exponential decay model fitted to the data such that


$$y∼e^{\left(a+b*e^{\left(x*(-c)\right)}\right),}\;$$

where *x* is the distance of each entry to the diagonal, *y* is the value of the entry (barcode sharing between a pair of windows), and *a*, *b*, and *c* are parameters that are estimated from the data. While *a* relates to read coverage and noise in barcode sharing and *b* relates to read coverage (both being positively correlated), *c* is inversely related to molecule length (supplementary fig. S12, Supplementary Material online).

The prediction bands fitted by the model include the background distribution of barcode sharing and any windows whose values are outside the prediction bands (*α* = 0.05) are then classified as putative SVs. Once the model has been fitted, Wrath outputs a list of putative SVs with their genomic coordinates and produces plots of the fitted model and identified outliers. The putative SVs are not classified into SV types and are intended to be used for prioritization processes before further exploration.

Additionally, Wrath scales barcode sharing values according to their distance to the diagonal using Z-scores and filters out any absolute Z-score larger than a given threshold (default 2). This additional filter reduces the number of false positives close to the diagonal, as the closer the genomic windows are, the more variation there is in their barcode sharing.

Wrath can be applied to single populations to visualize and explore putative SVs in each chromosome of the genome. It can also be applied to detect SVs in different populations separately, which can then easily be compared and scanned for overlaps using bedtools (see case-studies below).

### Analyses of *Heliconius* Data

Data from Meier et al. (2021) were used. First, reads were pre-processed, and adapters and low-quality ends were trimmed using TRIMMOMATIC (Bolger et al. 2014). Then, we mapped the reads to the respective reference genome, *H. erato* v1.0 (Nadeau et al. 2014) and *H. melpomene* v2.5 (Davey et al. 2016, 2017), using BWA-MEM (Li 2013) and marked PCR duplicates using the MarkDuplicates utility from Picard tools (broadinstitute.github.io/picard). Alignment (BAM) files were used as input for Wrath, which we ran separately for each scaffold in the reference genome with a window size of 10 kb. Reads with a mapping quality below 10 were filtered out by Wrath. To examine the color loci specifically, we ran Wrath with a window size of 1 kb and specifying the desired coordinates.

### Simulations of SVs in the *H. melpomene* Genome

We tested Wrath's accuracy with synthetic SV data. We generated a modified version of *H. melpomene* Hmel2.5 genome assembly carrying 710 SVs of known location and size using the script simversion.py (https://github.com/simonhmartin/simversion). Insertions, deletions, and inversions ranging in size from 1 to 100 kb (1, 5, 10, 50, and 100 kb) were distributed along each chromosome with the intervals between them randomly sampled from an exponential distribution with mean 1/3 Mb. This approach of sampling the intervals between variants avoids any overlaps between the simulated SVs, but it does not rule out the possibility of overlap between simulated SVs and real SVs in the resequenced individuals. The simulated insertions comprised randomly generated sequences. We then mapped the *H. melpomene supp. plesseni* samples from Meier et al. (2021) to the modified genome following the methods above. We then applied Wrath to these simulated data using two window sizes, 5 and 10 kb, and evaluated the results.

To evaluate the accuracy of the detection, we defined three measures: overlap, error, and sensitivity (supplementary figs. S3–S5, Supplementary Material online). First, we calculated the overlap between the simulated SV and the detected SV relative to the length of the simulated SV. We also defined a measure of error of detection of SVs in the simulated data. This error is calculated by adding up the absolute distances between detected SV breakpoints and simulated ones and scaled by window size. The error can be interpreted as the number of genomic windows between the SV breakpoint detected by Wrath and the simulated SV breakpoint. Once we have calculated these two metrics, we select detected SV with the maximum overlap and minimum scaled error for each simulated SV, also filtering for a minimum overlap of 0.9. Finally, we defined 3 error thresholds: 15, 20, and 25 genomic windows (supplementary fig. S5, Supplementary Material online) and calculated sensitivity (true positives/(true positives + false negatives)) for each type of SV.

### *H. misippus* Sampling

Samples were collected in multiple locations in Africa. Most sample bodies were preserved in 100% ethanol immediately after collection, with some exceptions that were air dried. Samples in ethanol were kept at room temperature for 2 mo and then stored at −80 °C. A small (1/8) piece of the thorax was used for sequencing.

### *H. misippus* Sequencing Library Construction

DNA extractions and haplotag libraries were prepared essentially as described in Meier et al. (2021), with the some modifications. Haplotag libraries were prepared in batches of 96 samples with DNA sample diluted to 0.15 ng/µl with 10 mM Tris, pH 8, and quantified with Quant-iT PicoGreen dsDNA Assay Kit (Thermo Fisher Scientific). We used only 1.2 µl of haplotagging beads (∼0.88 million beads, each carrying one of 885K well-specific barcodes) per sample, 30 µl of WASH buffer (20 mM Tris pH 8, 50 mM NaCl, 0.1% Triton X-100), 10 µl of 5× tagmentation buffer (50 mM TAPS pH 8.5 with NaOH, 25 mM MgCl_2_, 50% N,N-dimethylformamide), and 25 µl of 0.6% SDS for Tn5-stripping following tagmentation. For sub-sampling, one-tenth of the beads + DNA (0.15 ng DNA per sample) from each of the 96 samples was pooled into a single eight-tube PCR strip, and then again from every eight pools into four final samples pools. With only four pooled samples on the magnetic stand, the buffer was removed, and 20 µl of 1× Lambda Exonuclease buffer, supplemented with 10 units of exonuclease and 5 units of Lambda exonuclease (New England BioLabs), was added to each sample. Samples were incubated at 37 °C for 30 min, and then washed twice for 5 min with 150 µl of WASH buffer. DNA library was then amplified using Q5 High-Fidelity DNA Polymerase (New England BioLabs) in four 25 µl PCR reactions according to manufacturer's instructions, using 4 µl of 10 µM TruSeq-F AATGATACGGCGACCACCGAGATCTACAC and TruSeq-R CAAGCAGAAGACGGCATACGAGAT primers, with the following cycling conditions: 10 min at 72 °C followed by 30 s 98 °C and 10 cycles of 98 °C for 15 s, 65 °C for 30 s, and 72 °C for 60 s. Libraries were pooled after PCR into a single library pool, size selected using Ampure magnetic beads (Beckman Coulter), Qubit quantified, and adjusted with 10 mM Tris, pH 8, 0.1 mM EDTA to 2.5 nM concentration for sequencing.

*Sequencing and demultiplexing*. Pooled libraries were sequenced by a HiSeq 3000 (Illumina) instrument at the Genome Core Facility at the MPI Tübingen Campus with a 150 + 13 + 12 + 150 cycle run setting, such that the run produced 13 and 12nt in the i7 and i5-index reads, respectively. Sequence data were first converted into fastq format using bcl2fastq v2.17.1.14 with the following parameters: –use-bases-mask = Y150, I13, I12, Y150; –minimum-trimmed-read-length = 1; –mask-short-adapter-reads = 1; –create-fastq-for-index-reads (Illumina).

### Quality Control and Read Mapping

First, molecules were demultiplexed. When using haplotagging, the molecule of origin information is embedded in the read name in the fastq file as a string of four barcodes of six nucleotides each. This was done as described in Meier et al. (2021) to generate the modified fastq files. The molecule ID is then included in the BX tag of each read. Barcode mismatches caused by sequencing errors are allowed as long as there is an unambiguous closest match. Once the BX tag was created, we pre-processed the reads, cutting adapters and low-quality ends using TRIMMOMATIC (Bolger et al. 2014), mapped to the reference genome HypMis_v2 using BWA-MEM2 (Vasimuddin et al. 2019), and sorted by coordinate using Samtools v1.9 (Danecek et al. 2021). PCR duplicates were marked using the MarkDuplicates utility from Picard tools (broadinstitute.github.io/picard) with two specific options CREATE_INDEX = TRUE and READ_ONE_BARCODE_TAG = BX. We then demultiplexed the individuals using their barcodes and included their individual ID information in the read group field.

### SNP Calling and Imputation

SNPs were identified using the mpileup utility of bcftools v1.11 (Danecek et al. 2021), running each chromosome separately including the INFO/AD, AD, DP, DV, DPR, INFO/DPR, DP4, SP tags in the output (-a option), setting the minimum mapping quality to 10 (-q) and the minimum base quality to 20 (-Q), ignoring Read Group tags (–ignore-RG) and removing duplicates (-F 1024), and the optput directly piped to bcftools call using the alternative model for multiallelic and rare-variant calling (–multiallelic-caller), including only variants in the output (–variants-only) and the fields GQ and GP (-f GQ, GP). Then, using bcftools query (-f), we generated a file containing the chromosome, position, reference, and alternative alleles for each SNP and with that produced a file of SNP positions that we could use as one of the inputs for the SNP imputation program STITCH (Davies et al. 2016). Following that, we generated genomic windows of 500 kb using bedtools over which we could iterate to run the remainder of the pipeline.

We ran STITCH separately for each of the genomic intervals using all our bam files as input. STITCH imputes SNPs from read and linked-read information but requires fine tuning of the input parameters. To optimize the values, we tested multiple values and compared the results, evaluating their performance using the M locus following methods from Meier et al. (2021). SNPs at the M locus are expected to be 0/0 or 0/1 for *misippus* individuals and 1/1 for *inaria* individuals. Options that optimized the results were *K* = 30, method = diploid, nGen = 500, readAware = TRUE, keepInterimFiles = FALSE, shuffle_bin_radius = 500, expRate = 5, iSizeUpperLimit = 500,000, keepSampleReadsInRAM = TRUE, and use_bx_tag = FALSE. We concatenated the resulting imputed variant calls (vcf files) using bcftools concat.

### SNP Phasing in *H. misippus*

To phase the SNPs into haplotypes, we used HapCut2 (Edge et al. 2017), which we ran separately for each of the 500 kb intervals used for imputation and each individual separately. First, we filtered SNPs based on their informativeness (INFO_SCORE ≥ 0.2) and selected all heterozygous SNPs. We used this as input for the –extractHAIRS utility of HapCut2 together with the BAM files with marked duplicates and the option –10× turned on, which indicates that the input contains linked-reads. This produced a file with unlinked fragments, which we then used as input for the LinkFragments.py script of HapCut2, which integrates the information of the linked-reads. We specified a maximum distance of 50 kb. Then, we used the linked fragment file and vcf as input for the HAPCUT2 utility with the option –nf 1 –threshold 30 –error_analysis_mode 1 –call_homozygous 1. Finally, we integrated the resulting vcf to our vcf of homozygous sites.

### Phenotyping and GWAS

We photographed forewings and hindwings of each individual in a standardized set-up, using a green background and a color checker. Phenotypes were scored by hand following the phenotype categorizations of Gordon et al. (2010), coding *misippus* morphs as 1, and *inaria* as 0. All sample phenotypes are found in supplementary table S2, Supplementary Material online. Using the merged HAPCUT2 output vcf file as input and the phenotype scores, we performed a GWAS with Plink v1.9 (Purcell et al. 2007) using the option –assoc.

### Detection of SVs in *H. misippus*

Genome wide SVs were identified using Wrath using the same method as for the *Heliconius* data. We used the intersect utility from bedtools v2.30.0 to assess overlap between SVs identified in homozygous recessive, and heterozygous and homozygous dominant individuals, setting the minimum fraction of overlap to 0.8 for both sets and extracting only one match per SV (intersectBed with options -f 0.8 -F 0.8 -u).

### DNA Extractions and Amplification of the M Locus in *H. misippus*

DNA extractions were carried out using a custom protocol using PureLink buffers and homemade magnetic beads. Briefly, a small piece of thorax tissue (1/10) is placed in a eight-tube PCR strip. Then, 45 ul of PureLink Digestion buffer and 10 ul of Proteinase K (20 mg/ml) are added, and the mix is incubated at 58 °C with shaking (500 rpm). Thereafter, we added 2 ul of RNAseA (DNAse free, 10 mg/ml) and incubated it 10 min at room temperature. Then, we added 45 ul of PureLink Lysis buffer and incubated at 58 °C for 30 min with shaking (500 rpm). We then used a homemade magnetic bead mix to extract the DNA from the lysate. First, we added 37.5 ul of magnetic beads and 75 ul of lysate to a 96-well plate. After mixing, we incubated for 15 min at room temperature, placed the plate in a magnetic stand for 10 min, removed the supernatant and cleaned the beads with 80% ethanol. After drying out, we added 50 ul of 10 mM Tris (pH = 8) to elute and incubated at 45 °C for 15 min without resuspending. Then, we resuspended the beads and incubated for 20 min at room temperature. Finally, we placed the plate on the magnetic stand and, after 10 min, transferred the supernatant (the DNA) to a fresh tube.

To amplify the regions of interest, we designed primers at each side of the deletions and at the breakpoints (supplementary fig. S11, Supplementary Material online). We used a Q5 High-Fidelity 2× Master Mix from New England BioLabs and with 35 cycles. We used eight individuals: four *inaria/immima* (CAM035232, CAM035239, CAM035244, and CAM035250) and four *misippus* (CAM035230, CAM035240, CAM035245, and CAM035249).

### Reference Genome Alignments

To identify putative homologous regions of the reference genomes of *H. bolina* HypBol_v1 (Orteu et al. 2023) and *H misippus*, we aligned the two references to each using Satsuma2 (Grabherr et al. 2010) with default parameters. We visualized the resulting alignments using the asynt R functions (Kim et al. 2022) from https://github.com/simonhmartin/asynt.

### Sample Preparation and Genome Wide Analysis of *H. bolina* Samples and Other *Hypolimnas* Species

A total of 214 wild and reared *H. bolina* samples were used. Briefly, sample DNA was extracted, and DNA Nextera libraries were prepared using custom protocols. First, DNA was extracted of the *H. bolina* samples following a custom protocol that uses PureLink buffers and homemade magnetic beads. Briefly, a small piece of thorax tissue was placed in PureLink Digestion buffer and Proteinase K and incubated for 2–3 h at 58 °C with shaking (500 rpm). RNAse (DNAse free) was added together with PureLink Lysis buffer and the samples were incubated for 30 min at 58 °C with shaking (500 rpm). Afterwards, to pellet any undigested solids, the samples were spun at 4,000*g* for 10 min at room temperature. Following that, the DNA was extracted from the lysate using a homemade magnetic bead mix with two rounds of 80% ethanol clean-ups.

From the extracted DNA, libraries were prepared following a method based on Nextera DNA Library Prep (Illumina, Inc.) with purified Tn5 transposase (Picelli et al. 2014). PCR extension with the N701–N800 i7-index primer and the N501–N508 and N5017 i5-index primers was performed to barcode the samples. Library purification and size selection were done using the same homemade beads as above.

Short-read data from whole genomes were sequenced to ∼6.5× in coverage. Reads were trimmed using fastp (Chen et al. 2018) and mapped to the reference genome HypMis_v2 using BWA-MEM2 (Vasimuddin et al. 2019) and sorted by coordinate using Samtools v1.9 (Danecek et al. 2021). PCR duplicates were marked using MarkDuplicatesSpark from GATK (Auwera and O’Connor 2020) and SNPs called bcftools v1.11 (Danecek et al. 2021) mpileup like described above. Imputation was carried out with STITCH (Davies et al. 2016) using the same settings as for *H. misippus*.

### Read Depth Analysis and PCA

We calculated read depth from the bam files using the depth utility from samtools (Danecek et al. 2021) with the –a option to output depth for all sites, including those with no reads mapping to them. We visualized the output in R v 4.1.2 using the ggplot2 package (Wickham 2016). VCFs of phased haplotypes were used for PCA, which was performed using Plink v1.9. For local PCA, a sliding window of 25 SNPs was used.

## Supplementary Material

msae041_Supplementary_Data

## Data Availability

Wrath is available at https://github.com/annaorteu/wrath. Whole genome sequencing samples of *H. misippus*, *H. bolina*, and other *Hypolimnas* are available at ENA project accession PRJEB64669. Supporting Supplementary Material online including *H. erato* and *H. melpomene* and *H. misippus* Wrath output and *H. melpomene* simulation results have been deposited in dryad 10.5281/zenodo.8199940.
