# Supplementary material for: Transposable Element Insertions Are Associated with Batesian Mimicry in the Pantropical Butterfly Hypolimnas misippus
Source: Mol Biol Evol. 2024 Feb 24;41(3):msae041. doi: 10.1093/molbev/msae041 (PMC10924252; doi:10.1093/molbev/msae041)
Supplement: msae041_Supplementary_Data [file msae041_supplementary_data.pdf]

Supplementary Information for

Transposable element insertions are associated with Batesian mimicry in the pantropical butterfly *Hypolimnas misippus*

Supplementary Figures

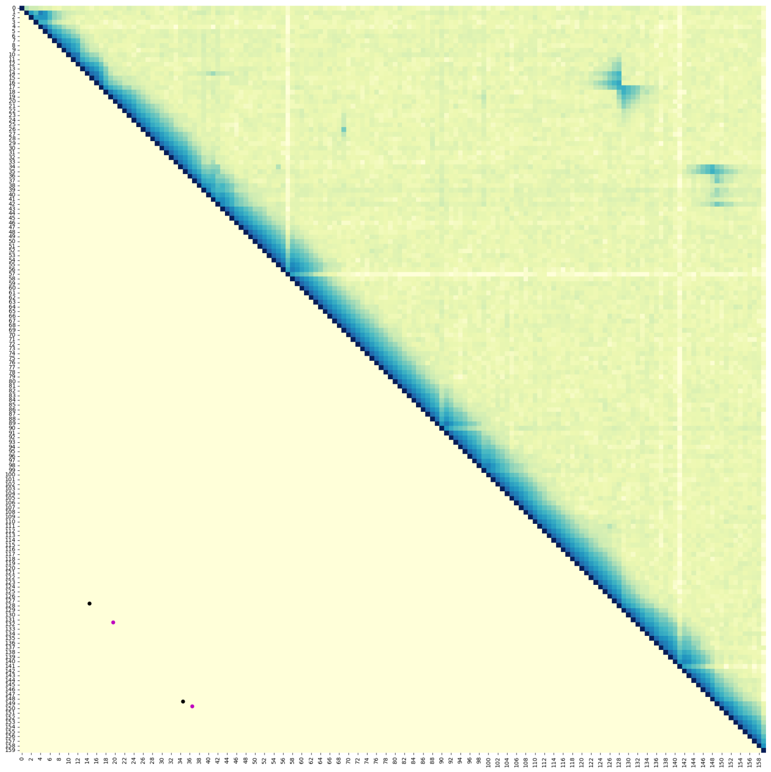

Supplementary Figure 1. Wrath output of scaffold Herato0204 in 10 kb windows identifies a known inversion. A bowtie pattern of barcode sharing can be observed off the diagonal. All *Heliconius erato* samples from Meier, et al., 2021, were used for the analysis.

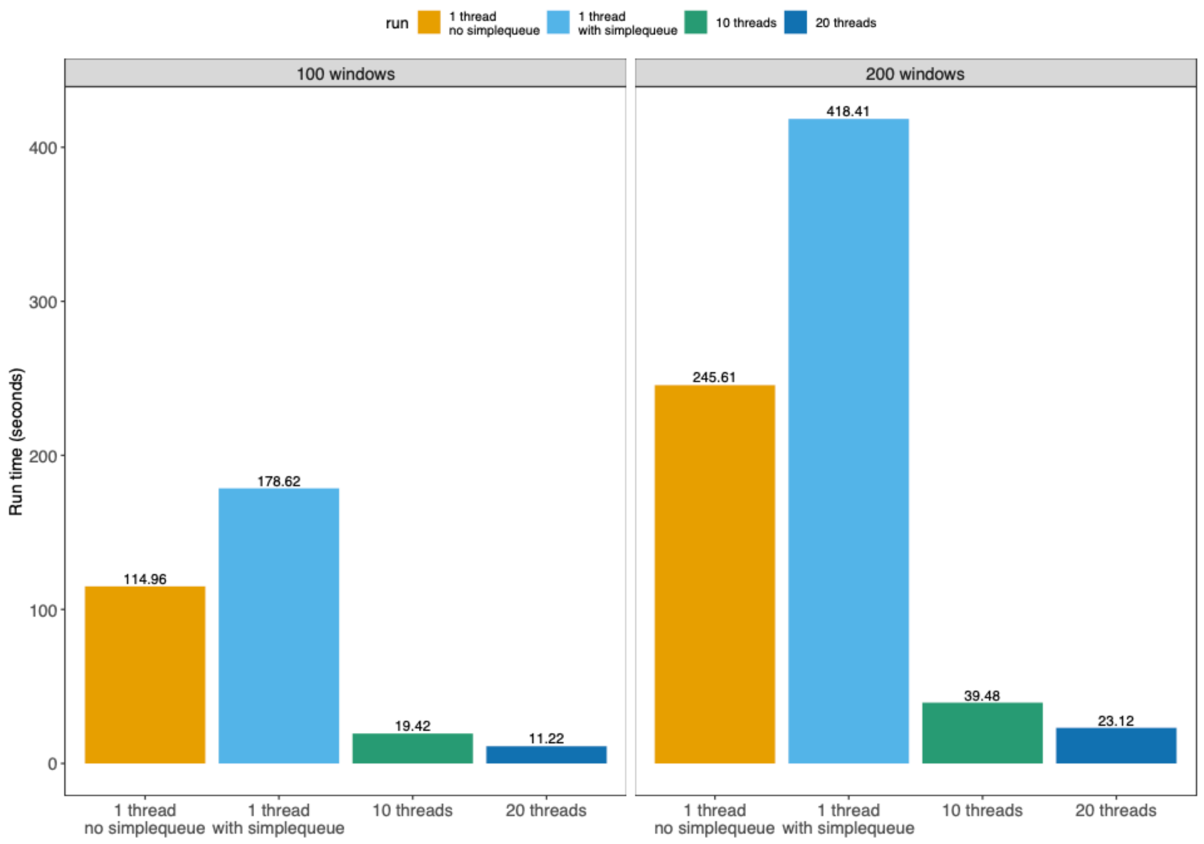

Supplementary Figure 2. Performance tests show that Wrath is 10x faster with 20 threads than with a single thread. Performance tests were performed using a subset of genomic windows (100 and 200). 4 scenarios were compared and the run time calculated. To implement multithreading Simplequeue in Python3 was used. A single threaded implementation of Wrath without Simplequeue ran faster than a single thread run with Simplequeue. However, increasing the number of threads decreases run time considerably.

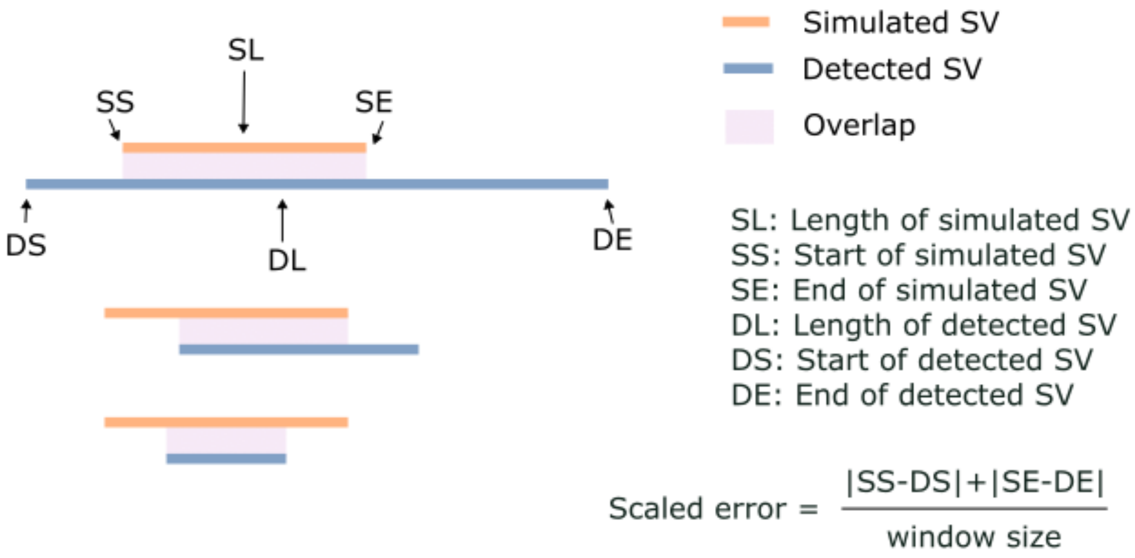

Supplementary Figure 3. Measure of error of Wrath at detecting SVs from simulated data. The error is calculated by the summation of the absolute distance between the identified breakpoints and the simulated ones. The error is scaled by window size to make it easier to interpret. On the left, three possible scenarios are depicted.

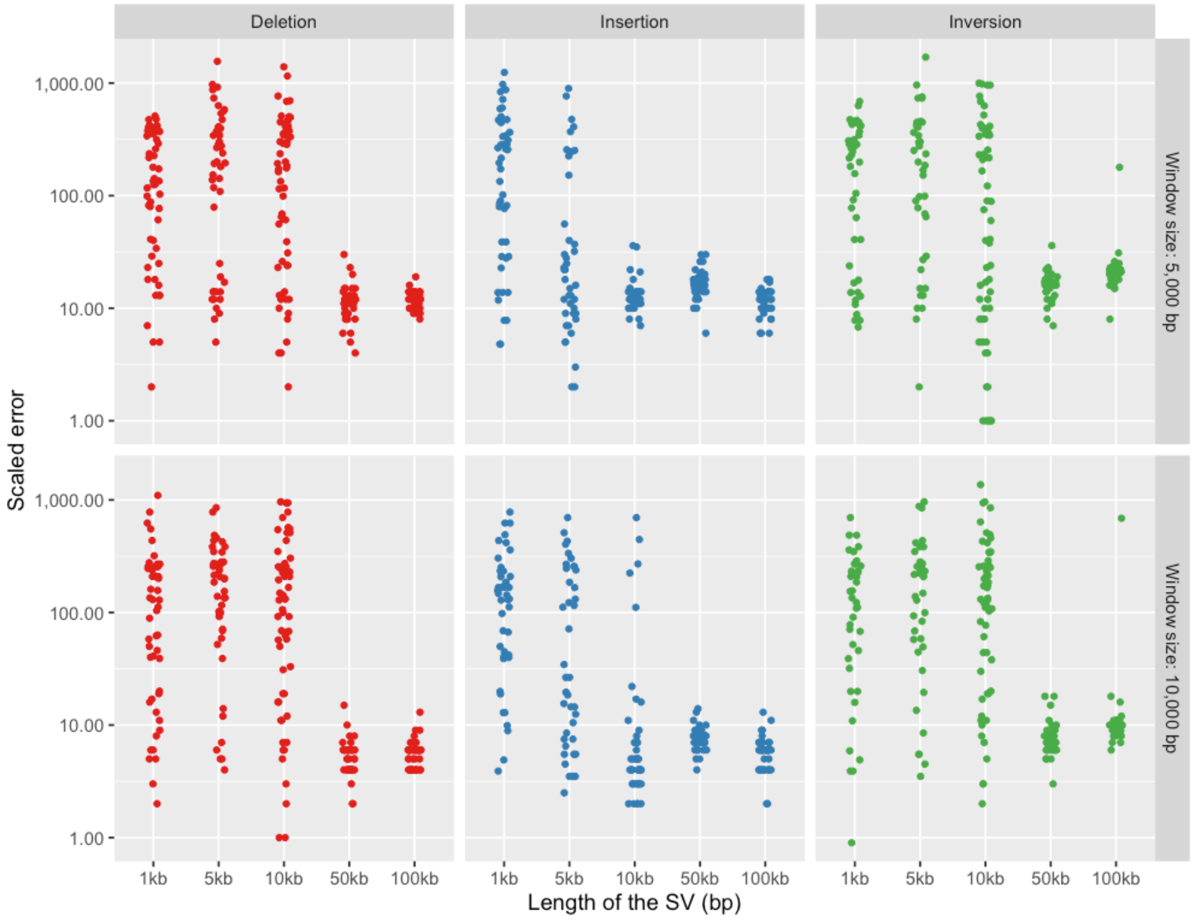

Supplementary Figure 4. Detectability of simulated SVs of different sizes using two distinct genomic window sizes (5kb and 10kb). Results are similar in both cases. However, given that the error is scaled by window size, absolute distance between the detected and simulated breakpoints is smaller with 5kb windows than with 10kb. Molecule size (40-60kb in this dataset) and window size limit the detectability of inversions and deletions, as barcode sharing is homogeneous across the SV if molecule size is much larger than the SV size and the pattern of barcode sharing is diffused if window size is similar to SV size. This is not the case for insertions as these always leave a signal of absence of barcode sharing (the same pattern left by translocations shown in the fourth column of Figure 1A-C); their detectability is mostly limited by window size with respect to SV size.

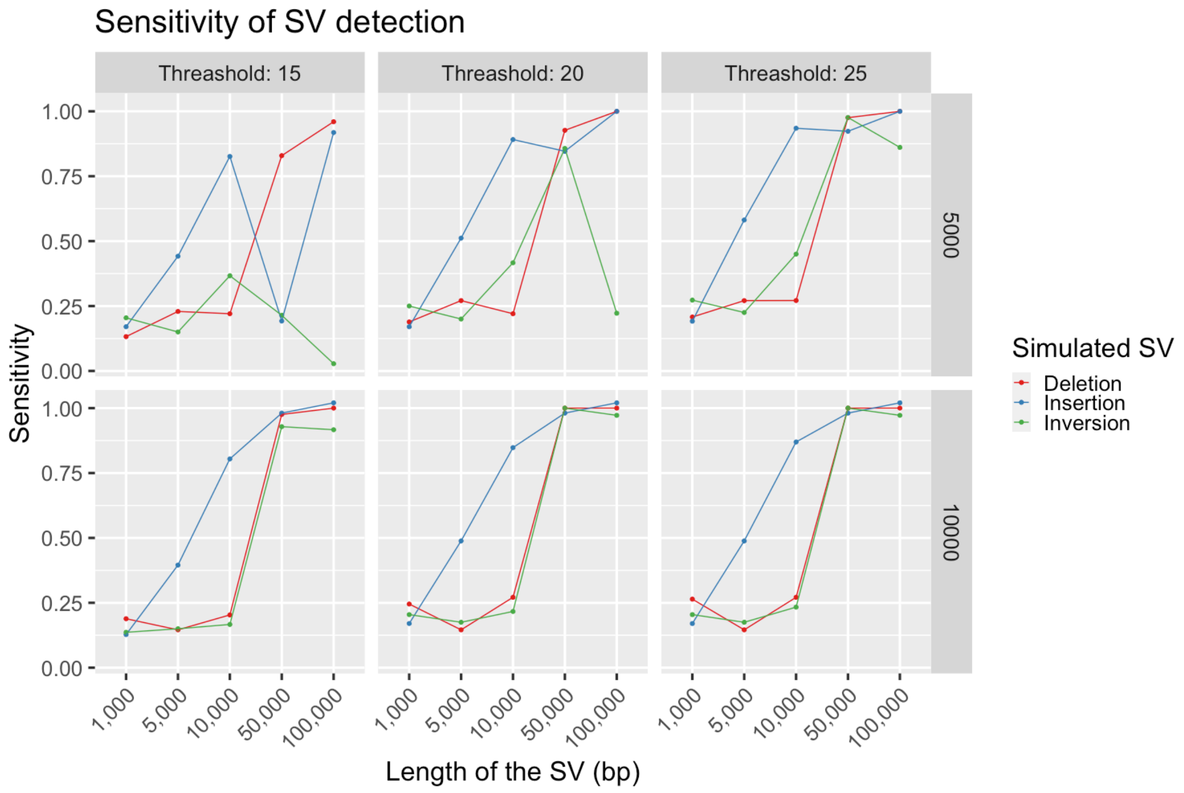

Supplementary Figure 5. Sensitivity of SV detection when using different genomic window sizes (5kb and 10kb) and applying 3 distinct scaled error thresholds: 15, 20 and 25.

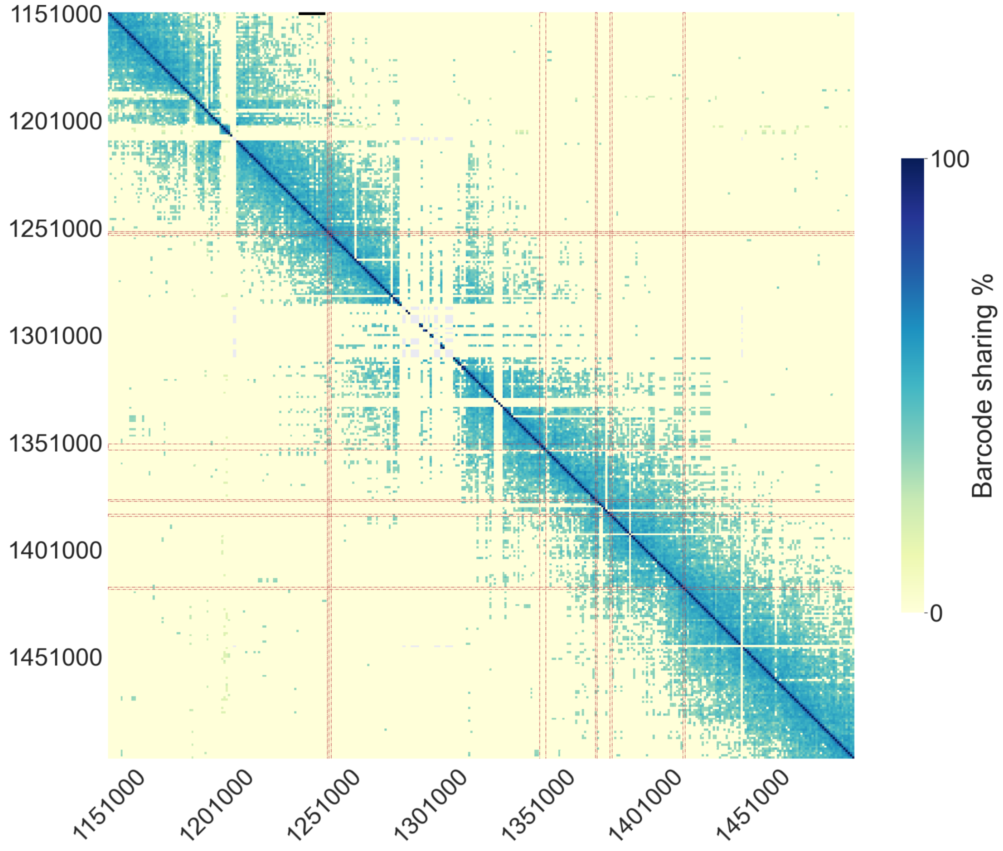

Supplementary Figure 6. Wrath output of the region around one of the loci (the *optix* locus) associated with colour pattern in *H. erato.* Each triangle half of the matrix depicts barcode sharing for one of the colour pattern subspecies, H. erato notabilis on top and H. erato lativitta at the bottom. Red dashed rectangles indicate the location of cis-regulatory elements indentified by Lewis et al. 2019 (from left to right, U1: 1252700-1252950, U2: 1253700-1254200, Obs132: 1351800-1354600, LR1: 1377700-1379000, LR2: 1384500-1386000, Obs214: 1418800-1419700). The black rectangle depicts the location of *optix.*

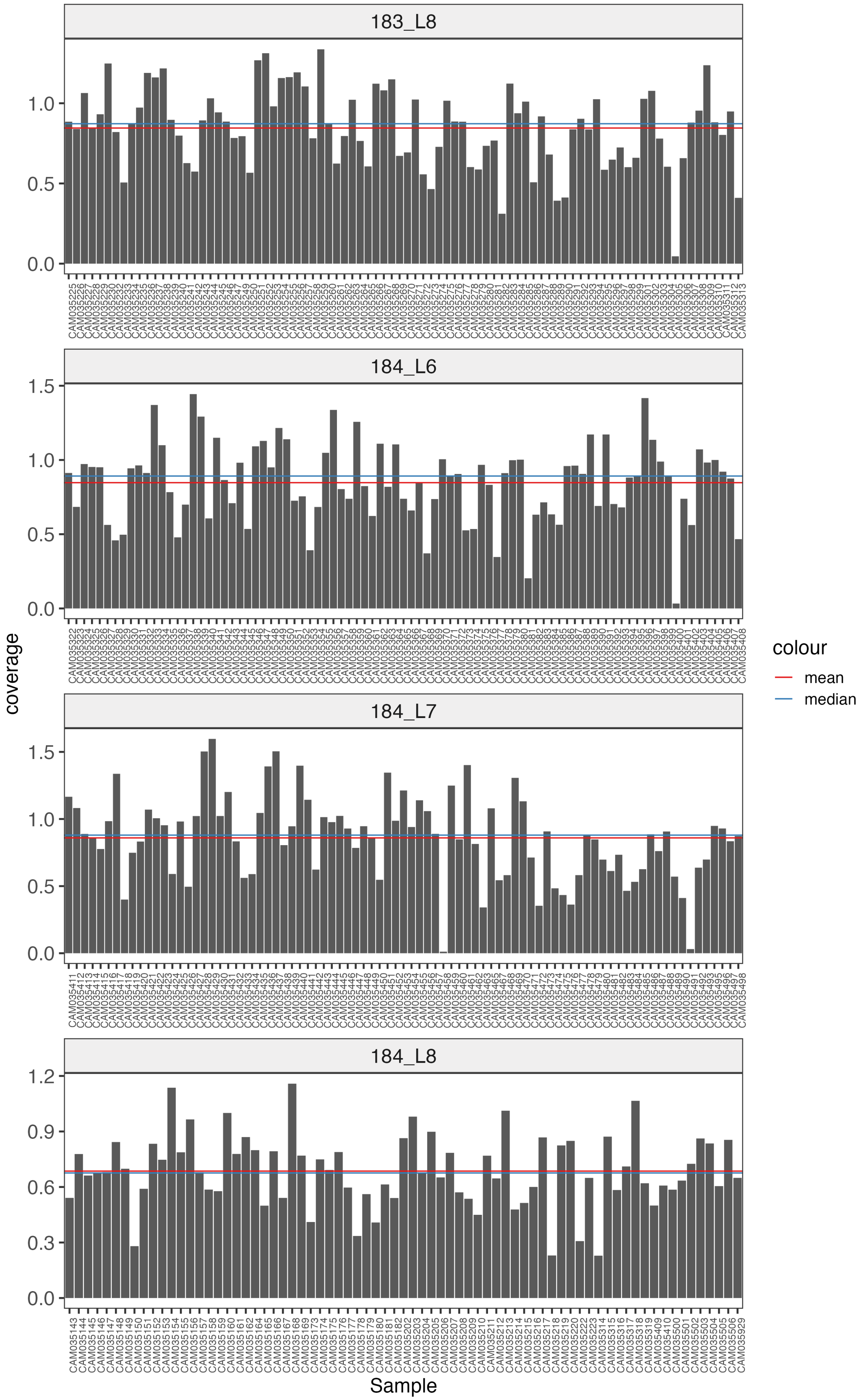
183_L8
184_L6
184_L7
184_L8
coverage
Sample
0.0
0.5
1.0
1.5
0.3
0.6
0.9
1.2
colour
mean
median

Supplementary Figure 7. Read coverage of *Hypolimnas misippus* haplotagging samples per barcode. *H. misippus* individuals were sequenced in four lanes, here shown each in a plot. Mean and median coverages per lane are shown in red and blue respectively.

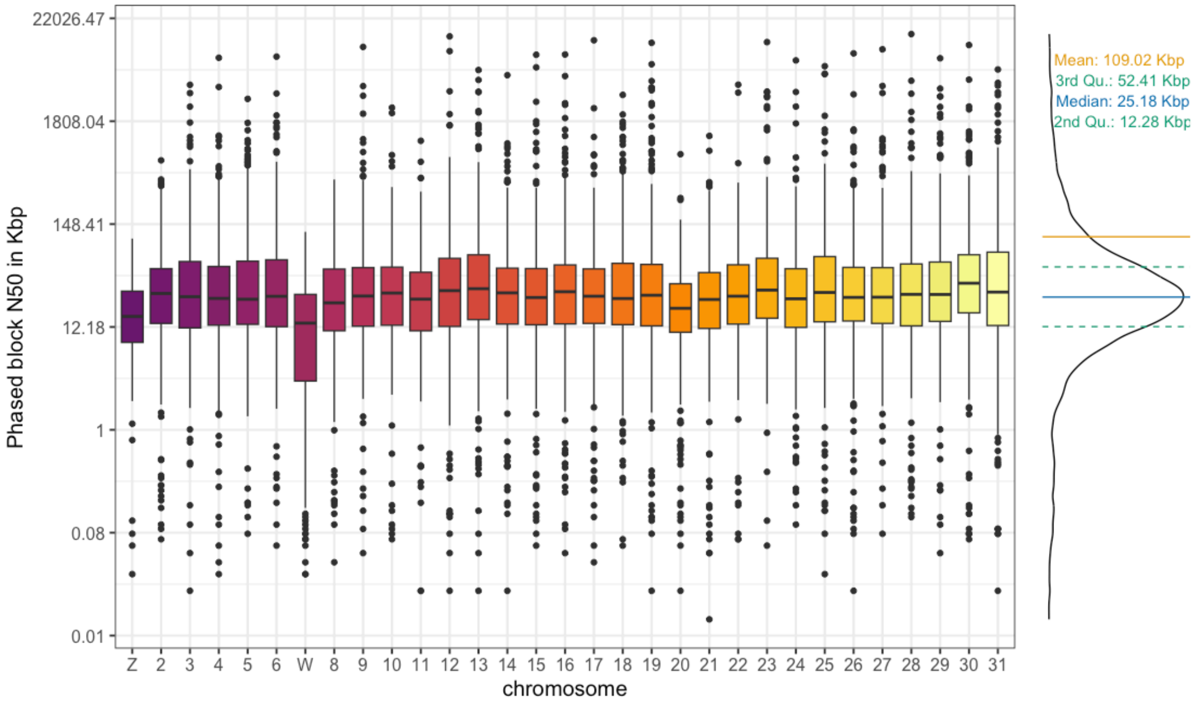

Supplementary Figure 8. Phased block N50 (log) by chromosome of *H. misippus* samples after phasing with HAPCUT2 (Edge et al., 2017)

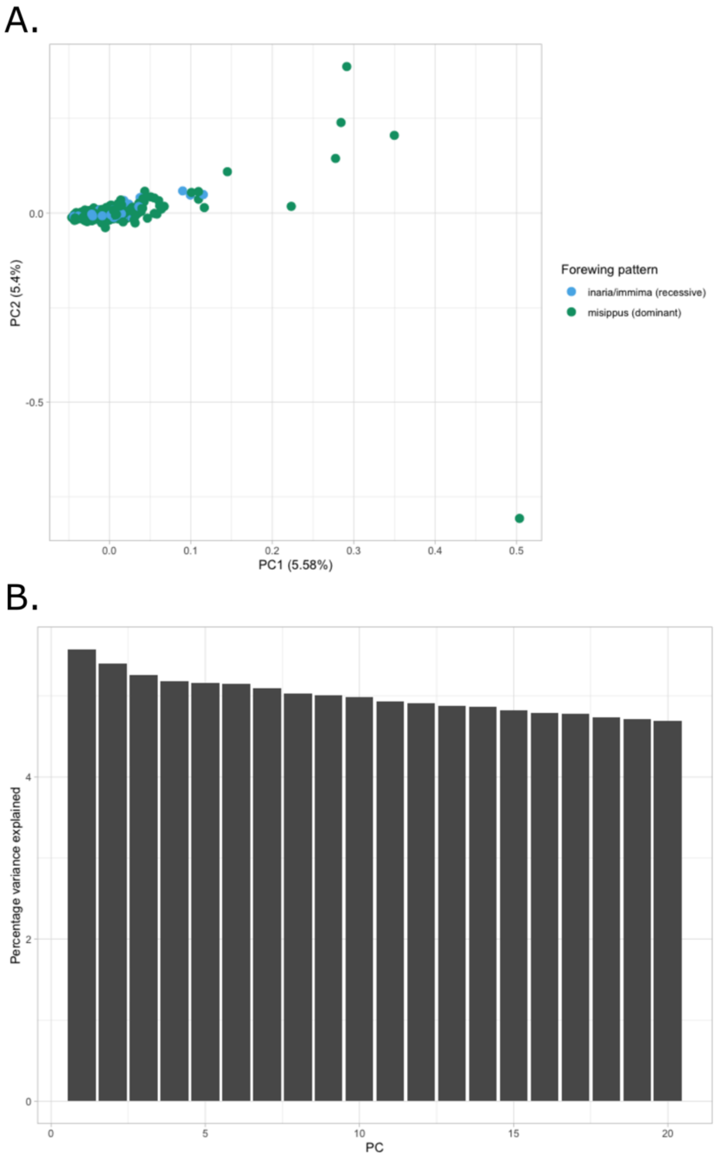

Supplementary Figure 9. Principal component Analysis (PCA) of *H. misippus* samples using the whole chromosome 29. PCA of the whole chromosome 29 reveals no structure by forewing phenotype (top). The percentage of variance explained by each principal component is low and constant, consistent with the absence of population structure (bottom).

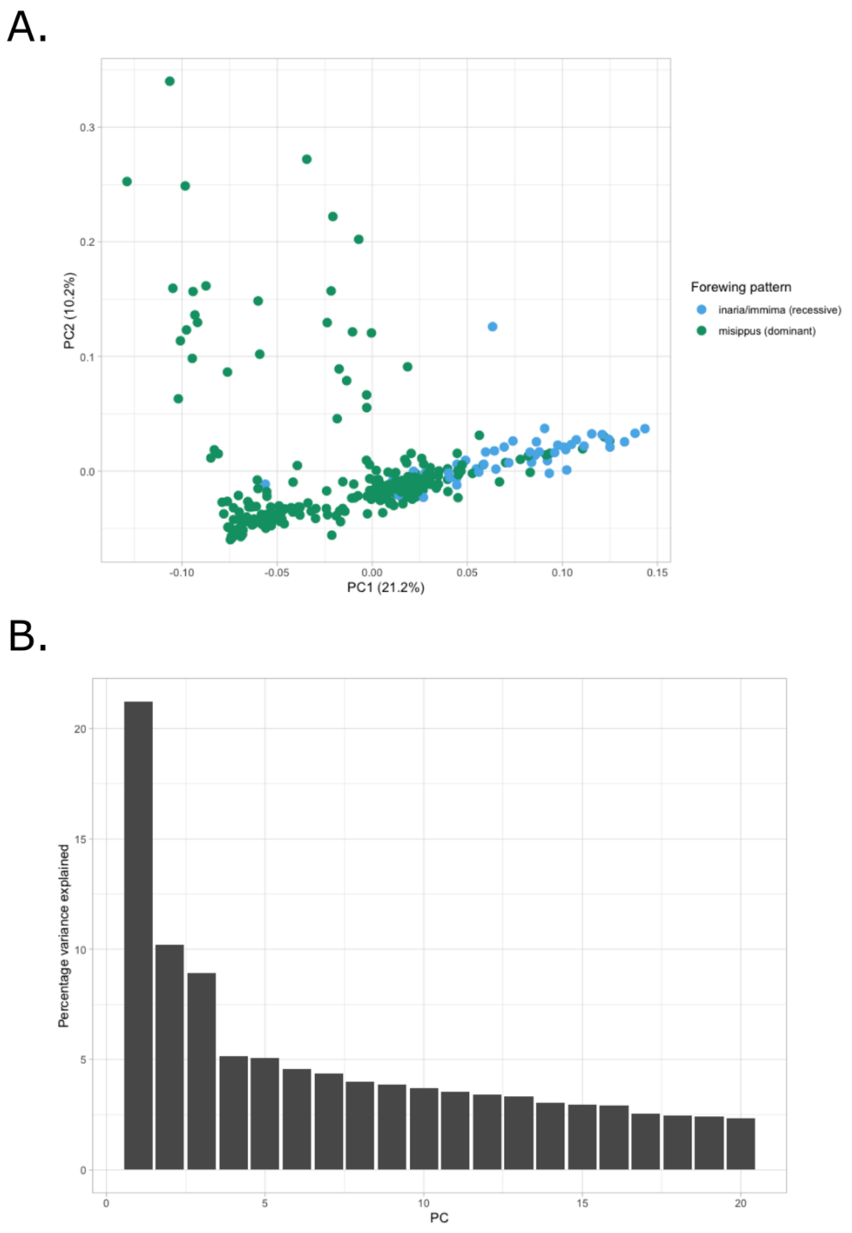

Supplementary Figure 10. Principal component Analysis (PCA) of *H. misippus* samples using the region associated with forewing phenotype. PCA of the region associated with forewing phenotype reveals structure by forewing phenotype (top). The percentage of variance explained by each principal component is higher for the first three components, consistent with the presence of population structure (bottom).

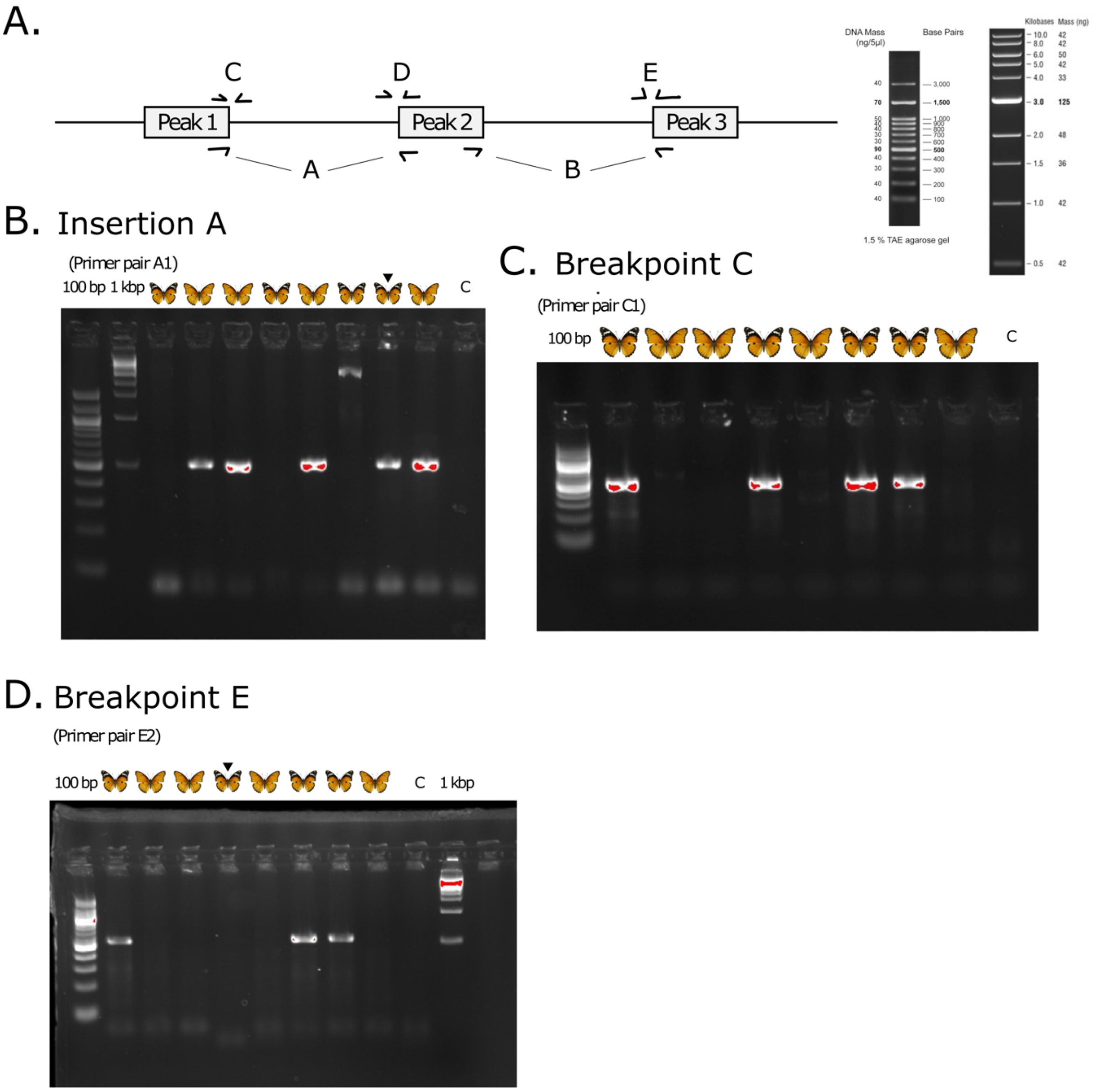

Supplementary Figure 11. PCR of the insertions at the locus associated with forewing phenotype. A. Diagram of the region associated with forewing phenotype with drawings of the primers used and ladders used in PCRs. B. PCR of insertion A shows that *mm* individuals (all orange forewings) have no insertion, as the PCR product is small (500 bp). One *M-* (Sample 7; black arrowhead) is heterozygote and thus also presents a band. C. PCR of breakpoint C shows that *M-* individuals possess insertion A, while *mm* do not. D. PCR of breakpoint E shows that *M-* individuals possess insertion B, while *mm* do not. No product was amplified from Sample 4 (*M-;* black arrowhead), possibly because of the high repeat and TE content. No PCR product was amplified at breakpoint D and insertion B.

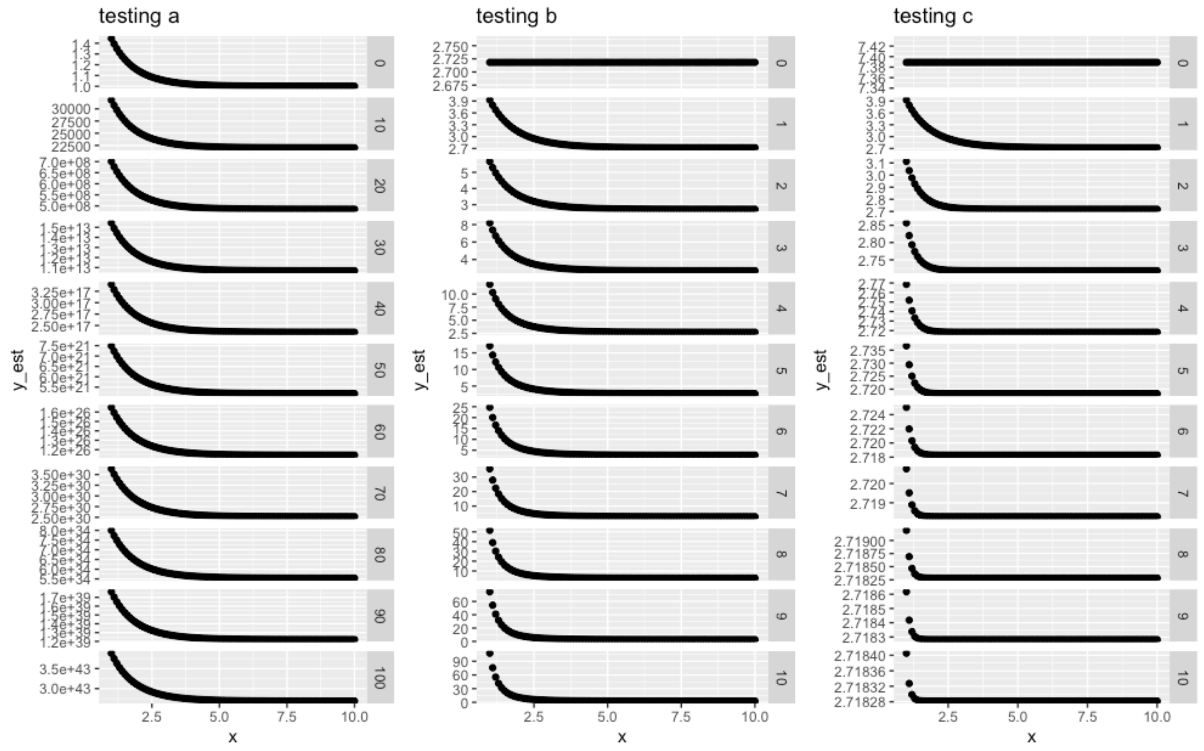

Supplementary Figure 12. Tests for a range of values of the *a, b,* and *c* parameters of the double exponential decay model fit by Wrath and used to detect outliers. Parameters are estimated from the data. *a* relates to read coverage and noise in barcode sharing. Large values of *a* indicate large amount of barcode sharing throughout the matrix, with neighbouring windows having higher barcode sharing, but also distant ones. The model tends asymptotically to higher values, the larger *a* is. *b* relates to read coverage only, influenced by the amount of barcode sharing of neighbouring windows, but not the overall amount like *a.* Datasets with higher coverage will produce higher values of *b.* Finally, *c* relates to molecule length. The longer the molecule, the smaller the value of c, with a minimum at 0, where molecules are as long as the chromosome, that is all genomic window comparisons have the same barcode sharing.

Supplementary Tables

Supplementary Table 1: Information on the samples collected and sequenced.

CAMID	Date	Location
**CAM035143**	23/04/2019	Kenya
**CAM035144**	24/04/2019	Kenya
**CAM035145**	16/10/2019	Kenya
**CAM035146**	16/10/2019	Kenya
**CAM035147**	16/10/2019	Kenya
**CAM035148**	16/10/2019	Kenya
**CAM035149**	16/10/2019	Kenya
**CAM035150**	16/10/2019	Kenya
**CAM035151**	16/10/2019	Kenya
**CAM035152**	16/10/2019	Kenya
**CAM035153**	16/10/2019	Kenya
**CAM035154**	24/04/2019	Kenya
**CAM035155**	24/04/2019	Kenya
**CAM035156**	25/04/2019	Kenya
**CAM035157**	25/04/2019	Kenya
**CAM035158**	25/04/2019	Kenya
**CAM035159**	26/04/2019	Kenya
**CAM035160**	29/04/2019	Kenya
**CAM035161**	28/04/2019	Kenya
**CAM035162**	28/04/2019	Kenya
**CAM035164**	28/04/2019	Kenya
**CAM035165**	28/04/2019	Kenya
**CAM035166**	30/04/2019	Kenya
**CAM035167**	28/04/2019	Kenya
**CAM035168**	28/04/2019	Kenya
**CAM035169**	30/04/2019	Kenya
**CAM035173**	01/12/2017	Mpala Research Centre, Laikipia, Kenya
**CAM035174**	09/12/2017	Bulisa, Uganda
**CAM035175**	NA	NA
**CAM035176**	24/04/2014	Tanzania
**CAM035177**	04/10/2012	forêt de Houéyogbé, Houéyogbé, Bénin
**CAM035178**	31/12/2010	forêt de Houéyogbé, Houéyogbé, Bénin
**CAM035179**	04/01/2010	route de Cové, pont du Zou, Bohicon, Bénin
**CAM035180**	04/02/2012	forêt de Pobé, Pobé, Bénin
**CAM035181**	10/04/2012	forêt de Houéyogbé, Houéyogbé, Bénin
**CAM035182**	11/09/2011	forêt de Houéyogbé, Houéyogbé, Bénin
**CAM035202**	15/04/2019	Watamu, Kenya
**CAM035203**	15/04/2019	Watamu, Kenya

**CAM035204**	15/04/2019	Watamu, Kenya
**CAM035205**	15/04/2019	Watamu, Kenya
**CAM035206**	15/04/2019	Watamu, Kenya
**CAM035207**	15/04/2019	Watamu, Kenya
**CAM035208**	15/04/2019	Watamu, Kenya
**CAM035209**	15/04/2019	Watamu, Kenya
**CAM035210**	15/04/2019	Watamu, Kenya
**CAM035211**	15/04/2019	Watamu, Kenya
**CAM035212**	15/04/2019	Watamu, Kenya
**CAM035213**	15/04/2019	Watamu, Kenya
**CAM035214**	15/04/2019	Watamu, Kenya
**CAM035215**	15/04/2019	Watamu, Kenya
**CAM035216**	15/04/2019	Watamu, Kenya
**CAM035217**	15/04/2019	Watamu, Kenya
**CAM035218**	15/04/2019	Watamu, Kenya
**CAM035219**	15/04/2019	Watamu, Kenya
**CAM035220**	28/04/2019	Mpala, Laikipia, Kenya
**CAM035222**	06/05/2019	Rwanda
**CAM035223**	08/12/2018	Biakpa, Volta Region, Ghana
**CAM035225**	May-June 2019	Watamu, Kenya
**CAM035226**	May-June 2019	Watamu, Kenya
**CAM035227**	May-June 2019	Watamu, Kenya
**CAM035228**	May-June 2019	Watamu, Kenya
**CAM035229**	May-June 2019	Watamu, Kenya
**CAM035230**	May-June 2019	Watamu, Kenya
**CAM035232**	May-June 2019	Watamu, Kenya
**CAM035233**	May-June 2019	Watamu, Kenya
**CAM035234**	May-June 2019	Watamu, Kenya
**CAM035235**	May-June 2019	Watamu, Kenya
**CAM035236**	May-June 2019	Watamu, Kenya
**CAM035237**	May-June 2019	Watamu, Kenya
**CAM035238**	May-June 2019	Watamu, Kenya
**CAM035239**	May-June 2019	Watamu, Kenya
**CAM035240**	May-June 2019	Watamu, Kenya
**CAM035241**	May-June 2019	Watamu, Kenya
**CAM035242**	May-June 2019	Watamu, Kenya
**CAM035243**	May-June 2019	Watamu, Kenya
**CAM035244**	May-June 2019	Watamu, Kenya
**CAM035245**	May-June 2019	Watamu, Kenya
**CAM035246**	May-June 2019	Watamu, Kenya
**CAM035247**	May-June 2019	Watamu, Kenya
**CAM035248**	May-June 2019	Watamu, Kenya
**CAM035249**	May-June 2019	Watamu, Kenya

**CAM035250**	May-June 2019	Watamu, Kenya
**CAM035251**	May-June 2019	Watamu, Kenya
**CAM035252**	May-June 2019	Watamu, Kenya
**CAM035253**	May-June 2019	Watamu, Kenya
**CAM035254**	May-June 2019	Watamu, Kenya
**CAM035255**	May-June 2019	Watamu, Kenya
**CAM035256**	May-June 2019	Watamu, Kenya
**CAM035257**	May-June 2019	Watamu, Kenya
**CAM035258**	May-June 2019	Watamu, Kenya
**CAM035259**	May-June 2019	Watamu, Kenya
**CAM035260**	May-June 2019	Watamu, Kenya
**CAM035261**	May-June 2019	Watamu, Kenya
**CAM035262**	May-June 2019	Watamu, Kenya
**CAM035263**	May-June 2019	Watamu, Kenya
**CAM035264**	May-June 2019	Watamu, Kenya
**CAM035265**	May-June 2019	Watamu, Kenya
**CAM035266**	May-June 2019	Watamu, Kenya
**CAM035267**	May-June 2019	Watamu, Kenya
**CAM035268**	May-June 2019	Watamu, Kenya
**CAM035269**	May-June 2019	Watamu, Kenya
**CAM035270**	May-June 2019	Watamu, Kenya
**CAM035271**	May-June 2019	Watamu, Kenya
**CAM035272**	May-June 2019	Watamu, Kenya
**CAM035273**	May-June 2019	Watamu, Kenya
**CAM035274**	May-June 2019	Watamu, Kenya
**CAM035275**	May-June 2019	Watamu, Kenya
**CAM035276**	May-June 2019	Watamu, Kenya
**CAM035277**	May-June 2019	Watamu, Kenya
**CAM035278**	May-June 2019	Watamu, Kenya
**CAM035279**	May-June 2019	Watamu, Kenya
**CAM035280**	May-June 2019	Watamu, Kenya
**CAM035281**	May-June 2019	Watamu, Kenya
**CAM035282**	May-June 2019	Watamu, Kenya
**CAM035283**	May-June 2019	Watamu, Kenya
**CAM035284**	May-June 2019	Watamu, Kenya
**CAM035285**	May-June 2019	Watamu, Kenya
**CAM035286**	May-June 2019	Watamu, Kenya
**CAM035287**	May-June 2019	Watamu, Kenya
**CAM035288**	May-June 2019	Watamu, Kenya
**CAM035289**	May-June 2019	Watamu, Kenya
**CAM035290**	May-June 2019	Watamu, Kenya
**CAM035291**	May-June 2019	Watamu, Kenya
**CAM035292**	May-June 2019	Watamu, Kenya

**CAM035293**	May-June 2019	Watamu, Kenya
**CAM035294**	May-June 2019	Watamu, Kenya
**CAM035295**	May-June 2019	Watamu, Kenya
**CAM035296**	May-June 2019	Watamu, Kenya
**CAM035297**	May-June 2019	Watamu, Kenya
**CAM035298**	May-June 2019	Watamu, Kenya
**CAM035299**	May-June 2019	Watamu, Kenya
**CAM035300**	May-June 2019	Watamu, Kenya
**CAM035301**	May-June 2019	Watamu, Kenya
**CAM035302**	May-June 2019	Watamu, Kenya
**CAM035303**	May-June 2019	Watamu, Kenya
**CAM035304**	May-June 2019	Watamu, Kenya
**CAM035305**	May-June 2019	Watamu, Kenya
**CAM035306**	May-June 2019	Watamu, Kenya
**CAM035307**	May-June 2019	Watamu, Kenya
**CAM035308**	May-June 2019	Watamu, Kenya
**CAM035309**	May-June 2019	Watamu, Kenya
**CAM035310**	May-June 2019	Watamu, Kenya
**CAM035311**	May-June 2019	Watamu, Kenya
**CAM035312**	May-June 2019	Watamu, Kenya
**CAM035313**	May-June 2019	Watamu, Kenya
**CAM035314**	May-June 2019	Watamu, Kenya
**CAM035315**	May-June 2019	Watamu, Kenya
**CAM035316**	May-June 2019	Watamu, Kenya
**CAM035317**	May-June 2019	Watamu, Kenya
**CAM035318**	May-June 2019	Watamu, Kenya
**CAM035319**	May-June 2019	Watamu, Kenya
**CAM035321**	May-June 2019	Watamu, Kenya
**CAM035322**	May-June 2019	Watamu, Kenya
**CAM035323**	May-June 2019	Watamu, Kenya
**CAM035324**	May-June 2019	Watamu, Kenya
**CAM035325**	May-June 2019	Watamu, Kenya
**CAM035326**	May-June 2019	Watamu, Kenya
**CAM035327**	May-June 2019	Watamu, Kenya
**CAM035328**	May-June 2019	Watamu, Kenya
**CAM035329**	May-June 2019	Watamu, Kenya
**CAM035330**	May-June 2019	Watamu, Kenya
**CAM035331**	May-June 2019	Watamu, Kenya
**CAM035332**	May-June 2019	Watamu, Kenya
**CAM035333**	May-June 2019	Watamu, Kenya
**CAM035334**	May-June 2019	Watamu, Kenya
**CAM035335**	May-June 2019	Watamu, Kenya
**CAM035336**	May-June 2019	Watamu, Kenya

**CAM035337**	May-June 2019	Watamu, Kenya
**CAM035338**	May-June 2019	Watamu, Kenya
**CAM035339**	May-June 2019	Watamu, Kenya
**CAM035340**	May-June 2019	Watamu, Kenya
**CAM035341**	May-June 2019	Watamu, Kenya
**CAM035342**	May-June 2019	Watamu, Kenya
**CAM035343**	May-June 2019	Watamu, Kenya
**CAM035344**	May-June 2019	Watamu, Kenya
**CAM035345**	May-June 2019	Watamu, Kenya
**CAM035346**	May-June 2019	Watamu, Kenya
**CAM035347**	May-June 2019	Watamu, Kenya
**CAM035348**	May-June 2019	Watamu, Kenya
**CAM035349**	May-June 2019	Watamu, Kenya
**CAM035350**	May-June 2019	Watamu, Kenya
**CAM035351**	May-June 2019	Watamu, Kenya
**CAM035352**	May-June 2019	Watamu, Kenya
**CAM035353**	May-June 2019	Watamu, Kenya
**CAM035354**	May-June 2019	Watamu, Kenya
**CAM035355**	May-June 2019	Watamu, Kenya
**CAM035356**	May-June 2019	Watamu, Kenya
**CAM035357**	May-June 2019	Watamu, Kenya
**CAM035358**	May-June 2019	Watamu, Kenya
**CAM035359**	May-June 2019	Watamu, Kenya
**CAM035360**	May-June 2019	Watamu, Kenya
**CAM035361**	May-June 2019	Watamu, Kenya
**CAM035362**	May-June 2019	Watamu, Kenya
**CAM035363**	May-June 2019	Watamu, Kenya
**CAM035364**	May-June 2019	Watamu, Kenya
**CAM035365**	May-June 2019	Watamu, Kenya
**CAM035366**	May-June 2019	Watamu, Kenya
**CAM035367**	May-June 2019	Watamu, Kenya
**CAM035368**	May-June 2019	Watamu, Kenya
**CAM035369**	May-June 2019	Watamu, Kenya
**CAM035370**	May-June 2019	Watamu, Kenya
**CAM035371**	May-June 2019	Watamu, Kenya
**CAM035372**	May-June 2019	Watamu, Kenya
**CAM035373**	May-June 2019	Watamu, Kenya
**CAM035374**	May-June 2019	Watamu, Kenya
**CAM035375**	May-June 2019	Watamu, Kenya
**CAM035376**	May-June 2019	Watamu, Kenya
**CAM035377**	May-June 2019	Watamu, Kenya
**CAM035378**	May-June 2019	Watamu, Kenya
**CAM035379**	May-June 2019	Watamu, Kenya

**CAM035380**	May-June 2019	Watamu, Kenya
**CAM035381**	May-June 2019	Watamu, Kenya
**CAM035382**	May-June 2019	Watamu, Kenya
**CAM035383**	May-June 2019	Watamu, Kenya
**CAM035384**	May-June 2019	Watamu, Kenya
**CAM035385**	May-June 2019	Watamu, Kenya
**CAM035386**	May-June 2019	Watamu, Kenya
**CAM035387**	May-June 2019	Watamu, Kenya
**CAM035388**	May-June 2019	Watamu, Kenya
**CAM035389**	May-June 2019	Watamu, Kenya
**CAM035390**	May-June 2019	Watamu, Kenya
**CAM035391**	May-June 2019	Watamu, Kenya
**CAM035392**	May-June 2019	Watamu, Kenya
**CAM035393**	May-June 2019	Watamu, Kenya
**CAM035394**	May-June 2019	Watamu, Kenya
**CAM035395**	May-June 2019	Watamu, Kenya
**CAM035396**	May-June 2019	Watamu, Kenya
**CAM035397**	May-June 2019	Watamu, Kenya
**CAM035398**	May-June 2019	Watamu, Kenya
**CAM035399**	May-June 2019	Watamu, Kenya
**CAM035400**	May-June 2019	Watamu, Kenya
**CAM035401**	May-June 2019	Watamu, Kenya
**CAM035402**	May-June 2019	Watamu, Kenya
**CAM035403**	May-June 2019	Watamu, Kenya
**CAM035404**	May-June 2019	Watamu, Kenya
**CAM035405**	May-June 2019	Watamu, Kenya
**CAM035406**	May-June 2019	Watamu, Kenya
**CAM035407**	May-June 2019	Watamu, Kenya
**CAM035408**	May-June 2019	Watamu, Kenya
**CAM035409**	May-June 2019	Watamu, Kenya
**CAM035410**	May-June 2019	Watamu, Kenya
**CAM035411**	May-June 2019	Watamu, Kenya
**CAM035412**	May-June 2019	Watamu, Kenya
**CAM035413**	May-June 2019	Watamu, Kenya
**CAM035414**	May-June 2019	Watamu, Kenya
**CAM035415**	May-June 2019	Watamu, Kenya
**CAM035416**	May-June 2019	Watamu, Kenya
**CAM035417**	May-June 2019	Watamu, Kenya
**CAM035418**	May-June 2019	Watamu, Kenya
**CAM035419**	May-June 2019	Watamu, Kenya
**CAM035420**	May-June 2019	Watamu, Kenya
**CAM035421**	May-June 2019	Watamu, Kenya
**CAM035422**	May-June 2019	Watamu, Kenya

**CAM035423**	May-June 2019	Watamu, Kenya
**CAM035424**	May-June 2019	Watamu, Kenya
**CAM035425**	May-June 2019	Watamu, Kenya
**CAM035426**	May-June 2019	Watamu, Kenya
**CAM035427**	May-June 2019	Watamu, Kenya
**CAM035428**	May-June 2019	Watamu, Kenya
**CAM035429**	May-June 2019	Watamu, Kenya
**CAM035430**	May-June 2019	Watamu, Kenya
**CAM035431**	May-June 2019	Watamu, Kenya
**CAM035432**	May-June 2019	Watamu, Kenya
**CAM035433**	May-June 2019	Watamu, Kenya
**CAM035434**	May-June 2019	Watamu, Kenya
**CAM035435**	May-June 2019	Watamu, Kenya
**CAM035436**	May-June 2019	Watamu, Kenya
**CAM035437**	May-June 2019	Watamu, Kenya
**CAM035438**	May-June 2019	Watamu, Kenya
**CAM035439**	May-June 2019	Watamu, Kenya
**CAM035440**	May-June 2019	Watamu, Kenya
**CAM035441**	May-June 2019	Watamu, Kenya
**CAM035442**	May-June 2019	Watamu, Kenya
**CAM035443**	May-June 2019	Watamu, Kenya
**CAM035444**	May-June 2019	Watamu, Kenya
**CAM035445**	May-June 2019	Watamu, Kenya
**CAM035446**	May-June 2019	Watamu, Kenya
**CAM035447**	May-June 2019	Watamu, Kenya
**CAM035448**	May-June 2019	Watamu, Kenya
**CAM035449**	May-June 2019	Watamu, Kenya
**CAM035450**	May-June 2019	Watamu, Kenya
**CAM035451**	May-June 2019	Watamu, Kenya
**CAM035452**	May-June 2019	Watamu, Kenya
**CAM035453**	May-June 2019	Watamu, Kenya
**CAM035455**	May-June 2019	Watamu, Kenya
**CAM035456**	May-June 2019	Watamu, Kenya
**CAM035457**	May-June 2019	Watamu, Kenya
**CAM035458**	May-June 2019	Watamu, Kenya
**CAM035459**	May-June 2019	Watamu, Kenya
**CAM035460**	May-June 2019	Watamu, Kenya
**CAM035461**	May-June 2019	Watamu, Kenya
**CAM035462**	May-June 2019	Watamu, Kenya
**CAM035463**	May-June 2019	Watamu, Kenya
**CAM035465**	May-June 2019	Watamu, Kenya
**CAM035466**	May-June 2019	Watamu, Kenya
**CAM035467**	May-June 2019	Watamu, Kenya

**CAM035468**	May-June 2019	Watamu, Kenya
**CAM035469**	May-June 2019	Watamu, Kenya
**CAM035470**	May-June 2019	Watamu, Kenya
**CAM035471**	May-June 2019	Watamu, Kenya
**CAM035472**	May-June 2019	Watamu, Kenya
**CAM035473**	May-June 2019	Watamu, Kenya
**CAM035474**	May-June 2019	Watamu, Kenya
**CAM035475**	May-June 2019	Watamu, Kenya
**CAM035476**	May-June 2019	Watamu, Kenya
**CAM035477**	May-June 2019	Watamu, Kenya
**CAM035478**	May-June 2019	Watamu, Kenya
**CAM035479**	May-June 2019	Watamu, Kenya
**CAM035480**	May-June 2019	Watamu, Kenya
**CAM035481**	May-June 2019	Watamu, Kenya
**CAM035482**	May-June 2019	Watamu, Kenya
**CAM035483**	May-June 2019	Watamu, Kenya
**CAM035484**	May-June 2019	Watamu, Kenya
**CAM035485**	May-June 2019	Watamu, Kenya
**CAM035486**	May-June 2019	Watamu, Kenya
**CAM035487**	May-June 2019	Watamu, Kenya
**CAM035488**	May-June 2019	Watamu, Kenya
**CAM035489**	May-June 2019	Watamu, Kenya
**CAM035490**	May-June 2019	Watamu, Kenya
**CAM035491**	May-June 2019	Watamu, Kenya
**CAM035492**	May-June 2019	Watamu, Kenya
**CAM035493**	May-June 2019	Watamu, Kenya
**CAM035495**	May-June 2019	Watamu, Kenya
**CAM035496**	May-June 2019	Watamu, Kenya
**CAM035497**	May-June 2019	Watamu, Kenya
**CAM035498**	May-June 2019	Watamu, Kenya
**CAM035499**	May-June 2019	Watamu, Kenya
**CAM035500**	May-June 2019	Watamu, Kenya
**CAM035501**	May-June 2019	Watamu, Kenya
**CAM035502**	May-June 2019	Watamu, Kenya
**CAM035503**	May-June 2019	Watamu, Kenya
**CAM035504**	May-June 2019	Watamu, Kenya
**CAM035505**	May-June 2019	Watamu, Kenya
**CAM035506**	May-June 2019	Watamu, Kenya
**CAM035929**	13/07/2018	Nigeria

Supplementary Table 2. Phenotypes and origin of wild *H. misippus* samples.

335 samples were collected and processed but only 332 used for analyses, as phenotypes were missing for 3 individuals (CAM035183, CAM035184 and CAM035185).

Sample	Forewing phenotype (i=*immima/inaria*, m=*misippus)*	Immima (1) or inaria (0)
**CAM035143**	m	NA
**CAM035144**	m	NA
**CAM035145**	m	NA
**CAM035146**	m	NA
**CAM035147**	m	NA
**CAM035148**	i	0
**CAM035149**	m	NA
**CAM035150**	m	NA
**CAM035151**	m	NA
**CAM035152**	m	NA
**CAM035153**	m	NA
**CAM035154**	m	NA
**CAM035155**	i	1
**CAM035156**	m	NA
**CAM035157**	i	1
**CAM035158**	m	NA
**CAM035159**	m	NA
**CAM035160**	m	NA
**CAM035161**	m	NA
**CAM035162**	m	NA
**CAM035164**	m	NA
**CAM035165**	m	NA
**CAM035166**	i	1
**CAM035167**	m	NA
**CAM035168**	m	NA
**CAM035169**	m	NA
**CAM035173**	m	NA
**CAM035174**	m	NA
**CAM035175**	m	NA
**CAM035176**	m	NA
**CAM035177**	m	NA
**CAM035178**	m	NA
**CAM035179**	m	NA
**CAM035180**	m	NA
**CAM035181**	i	0
**CAM035182**	m	NA
**CAM035202**	i	1
**CAM035203**	i	0
**CAM035204**	m	NA
**CAM035205**	m	NA
**CAM035206**	i	0
**CAM035207**	m	NA
**CAM035208**	m	NA
**CAM035209**	m	NA
**CAM035210**	m	NA
**CAM035211**	m	NA
**CAM035212**	i	0
**CAM035213**	m	NA
**CAM035214**	m	NA
**CAM035215**	i	0
**CAM035216**	m	NA
**CAM035217**	m	NA
**CAM035218**	m	NA
**CAM035219**	m	NA
**CAM035220**	m	NA
**CAM035222**	i	1
**CAM035223**	m	NA
**CAM035225**	m	NA
**CAM035226**	m	NA
**CAM035227**	m	NA
**CAM035228**	m	NA
**CAM035229**	m	NA
**CAM035230**	m	NA
**CAM035232**	i	1
**CAM035233**	m	NA
**CAM035234**	m	
**CAM035235**	m	NA
**CAM035236**	m	NA
**CAM035237**	m	NA
**CAM035238**	m	NA
**CAM035239**	i	0
**CAM035240**	m	NA
**CAM035241**	m	NA
**CAM035242**	m	NA
**CAM035243**	m	NA
**CAM035244**	i	1
**CAM035245**	m	NA
**CAM035246**	m	NA
**CAM035247**	m	NA
**CAM035249**	m	NA
**CAM035250**	i	1
**CAM035251**	i	1
**CAM035252**	i	1
**CAM035253**	i	1
**CAM035254**	i	1
**CAM035255**	m	NA
**CAM035256**	i	1
**CAM035257**	m	NA
**CAM035258**	m	NA
**CAM035259**	m	NA
**CAM035260**	m	NA
**CAM035261**	m	NA

**CAM035262**	m	NA
**CAM035263**	m	NA
**CAM035264**	i	0
**CAM035265**	m	NA
**CAM035266**	m	NA
**CAM035267**	m	NA
**CAM035268**	m	NA
**CAM035269**	m	NA
**CAM035270**	m	NA
**CAM035271**	m	NA
**CAM035272**	m	NA
**CAM035273**	m	NA
**CAM035274**	i	1
**CAM035275**	i	1
**CAM035276**	m	NA
**CAM035277**	m	NA
**CAM035278**	m	NA
**CAM035279**	i	1
**CAM035280**	m	NA
**CAM035281**	m	NA
**CAM035282**	m	NA
**CAM035283**	m	NA
**CAM035284**	m	NA
**CAM035285**	i	1
**CAM035286**	m	NA
**CAM035287**	i	1
**CAM035288**	m	NA
**CAM035289**	m	NA
**CAM035290**	m	NA
**CAM035291**	m	NA
**CAM035292**	m	NA
**CAM035293**	i	1
**CAM035294**	m	NA
**CAM035295**	m	NA
**CAM035296**	m	NA
**CAM035297**	m	NA
**CAM035298**	m	NA
**CAM035299**	m	NA
**CAM035301**	m	NA
**CAM035302**	i	1
**CAM035303**	m	NA
**CAM035304**	m	NA
**CAM035305**	m	NA
**CAM035306**	i	1
**CAM035307**	m	NA
**CAM035308**	m	NA
**CAM035309**	m	NA
**CAM035310**	i	1
**CAM035311**	m	NA
**CAM035312**	i	1
**CAM035313**	m	NA
**CAM035314**	m	NA
**CAM035315**	m	NA
**CAM035316**	m	NA
**CAM035317**	m	NA

**CAM035318**	m	NA
**CAM035319**	m	NA
**CAM035321**	m	NA
**CAM035322**	m	NA
**CAM035323**	m	NA
**CAM035324**	i	1
**CAM035325**	i	1
**CAM035326**	m	NA
**CAM035327**	m	NA
**CAM035328**	i	1
**CAM035329**	m	NA
**CAM035330**	m	NA
**CAM035331**	m	NA
**CAM035332**	m	NA
**CAM035333**	m	NA
**CAM035334**	m	NA
**CAM035335**	m	NA
**CAM035336**	m	NA
**CAM035337**	m	NA
**CAM035338**	m	NA
**CAM035339**	m	NA
**CAM035340**	m	NA
**CAM035341**	m	NA
**CAM035342**	m	NA
**CAM035343**	m	NA
**CAM035344**	m	NA
**CAM035345**	m	NA
**CAM035346**	m	NA
**CAM035347**	m	NA
**CAM035348**	m	NA
**CAM035349**	m	NA
**CAM035350**	i	1
**CAM035351**	m	NA
**CAM035352**	m	NA
**CAM035353**	m	NA
**CAM035354**	m	NA
**CAM035355**	m	NA
**CAM035356**	m	NA
**CAM035357**	m	NA
**CAM035358**	m	NA
**CAM035359**	m	NA
**CAM035360**	m	NA
**CAM035361**	m	NA
**CAM035362**	m	NA
**CAM035363**	m	NA
**CAM035364**	m	NA
**CAM035365**	m	NA
**CAM035366**	m	NA
**CAM035367**	m	NA
**CAM035368**	m	NA
**CAM035369**	m	NA
**CAM035370**	m	NA
**CAM035371**	m	NA
**CAM035372**	m	NA
**CAM035373**	m	NA

**CAM035374**	m	NA
**CAM035375**	m	NA
**CAM035376**	m	NA
**CAM035377**	i	1
**CAM035378**	m	NA
**CAM035379**	i	1
**CAM035380**	m	NA
**CAM035381**	m	NA
**CAM035382**	m	NA
**CAM035383**	m	NA
**CAM035384**	m	NA
**CAM035385**	m	NA
**CAM035386**	i	1
**CAM035387**	m	NA
**CAM035388**	m	NA
**CAM035389**	m	NA
**CAM035390**	m	NA
**CAM035391**	m	NA
**CAM035392**	m	NA
**CAM035393**	m	NA
**CAM035394**	m	NA
**CAM035395**	m	NA
**CAM035396**	m	NA
**CAM035397**	m	NA
**CAM035398**	m	NA
**CAM035399**	m	NA
**CAM035400**	m	NA
**CAM035401**	m	NA
**CAM035402**	m	NA
**CAM035403**	m	NA
**CAM035404**	i	1
**CAM035405**	m	NA
**CAM035406**	m	NA
**CAM035407**	i	1
**CAM035408**	m	NA
**CAM035409**	m	NA
**CAM035410**	m	NA
**CAM035411**	m	NA
**CAM035412**	m	NA
**CAM035413**	m	NA
**CAM035414**	i	1
**CAM035415**	m	NA
**CAM035416**	m	NA
**CAM035417**	i	1
**CAM035418**	i	0
**CAM035419**	m	NA
**CAM035420**	m	NA
**CAM035421**	m	NA
**CAM035422**	i	1
**CAM035423**	m	NA
**CAM035424**	m	NA
**CAM035425**	m	NA
**CAM035426**	m	NA
**CAM035427**	m	NA
**CAM035428**	m	NA
**CAM035429**	m	NA
**CAM035430**	m	NA
**CAM035431**	m	NA
**CAM035432**	m	NA
**CAM035433**	m	NA
**CAM035434**	m	NA
**CAM035435**	m	NA
**CAM035436**	m	NA
**CAM035437**	m	NA
**CAM035438**	m	NA
**CAM035439**	i	0
**CAM035440**	m	NA
**CAM035441**	m	NA
**CAM035442**	m	NA
**CAM035443**	i	1
**CAM035444**	m	NA
**CAM035445**	m	NA
**CAM035446**	i	1
**CAM035447**	m	NA
**CAM035448**	m	NA
**CAM035449**	m	NA
**CAM035450**	m	NA
**CAM035451**	m	NA
**CAM035452**	m	NA
**CAM035453**	i	0
**CAM035454**	m	NA
**CAM035455**	m	NA
**CAM035456**	m	NA
**CAM035457**	m	NA
**CAM035458**	m	NA
**CAM035459**	m	NA
**CAM035460**	m	NA
**CAM035461**	m	NA
**CAM035462**	m	NA
**CAM035463**	m	NA
**CAM035465**	m	NA
**CAM035467**	m	NA
**CAM035468**	m	NA
**CAM035469**	m	NA
**CAM035470**	m	NA
**CAM035471**	m	NA
**CAM035472**	i	1
**CAM035473**	m	NA
**CAM035474**	i	1
**CAM035475**	m	NA
**CAM035476**	m	NA
**CAM035477**	m	NA
**CAM035478**	m	NA
**CAM035479**	m	NA
**CAM035480**	m	NA
**CAM035481**	m	NA
**CAM035482**	m	NA
**CAM035483**	m	NA
**CAM035484**	m	NA
**CAM035485**	i	0

**CAM035486**	m	NA
**CAM035487**	m	NA
**CAM035488**	m	NA
**CAM035489**	i	0
**CAM035490**	m	NA
**CAM035491**	m	NA
**CAM035492**	i	1
**CAM035493**	m	NA
**CAM035495**	m	NA
**CAM035496**	i	0
**CAM035497**	m	NA
**CAM035498**	i	0
**CAM035500**	m	NA
**CAM035501**	m	NA
**CAM035502**	m	NA
**CAM035503**	m	NA
**CAM035504**	m	NA
**CAM035505**	i	0
**CAM035506**	m	NA
**CAM035929**	i	1

Supplementary Table 3. Read coverage statistics per lane.

Lane	Mean coverage	Median	Standard deviation
183_L6	0.846	0.872	0.249
184_L6	0.847	0.892	0.270
184_L7	0.853	0.877	0.320
184_L8	0.676	0.675	0.206

Supplementary Table 4. BLAST results of genes around the region around the associated locus with forewing phenotype against *Drosophila melanogaster.*

H.misippus transcript	*D.melanogaster* protein	*D. melanogaster* prot. description	E-value	Start pos.	End pos.
g1717.t1	NP_609354.1	uncharacterized protein	0	6001632	6069730
g1717.t1	NP_725571.1	uncharacterized protein, isoform B	0	6001632	6069730
g1717.t1	NP_611119.1	uncharacterized protein, isoform A	0	6001632	6069730
g1719.t1	NP_649078.1	uncharacterized protein, isoform A	5.56E-59	6073069	6074392
g1719.t1	NP_001262027.1	uncharacterized protein, isoform B	5.56E-59	6073069	6074392
g1721.t1	NP_649853.1	uncharacterized protein, isoform A	4.68E-64	6086308	6100583
g1721.t1	NP_001262394.1	uncharacterized protein, isoform B	4.68E-64	6086308	6100583
g1723.t1	NP_609418.1	lipase 4, isoform A	1.75E-109	6107821	6110369
g1727.t1	NP_648319.1	uncharacterized protein, isoform A	5.28E-44	6119757	6124412
g1727.t1	NP_001137926.1	uncharacterized protein, isoform D	5.28E-44	6119757	6124412
g1729.t1	NP_610796.1	Cyp301a1	0	6128617	6145667
g1729.t1	NP_995803.1	Cyp49a1, isoform D	2.61E-155	6128617	6145667
g1729.t1	NP_610588.2	Cyp49a1, isoform A	2.61E-155	6128617	6145667
g1729.t1	NP_001246256.1	Cyp49a1, isoform E	2.61E-155	6128617	6145667
g1730.t1	NP_995803.1	Cyp49a1, isoform D	3.7E-166	6158862	6200842

g1730.t1	NP_610588.2	Cyp49a1, isoform A	3.7E-166	6158862	6200842
g1730.t1	NP_001246256.1	Cyp49a1, isoform E	3.7E-166	6158862	6200842
g1730.t1	NP_610796.1	Cyp301a1	1.53E-154	6158862	6200842
g1731.t1	NP_649220.1	zye	0	6190393	6207540
g1731.t1	NP_649220.1	zye	8.13E-45	6190393	6207540
g1732.t1	NP_001137909.1	down syndrome cell adhesion molecule 4, isoform H	5.58E-35	6294473	6308283
g1732.t1	NP_001137909.1	down syndrome cell adhesion molecule 4, isoform H	4.7E-31	6294473	6308283
g1732.t1	NP_001137909.1	down syndrome cell adhesion molecule 4, isoform H	2.87E-16	6294473	6308283
g1732.t1	NP_001137908.1	down syndrome cell adhesion molecule 4, isoform D	7.04E-35	6294473	6308283
g1732.t1	NP_001137908.1	down syndrome cell adhesion molecule 4, isoform D	6.61E-31	6294473	6308283
g1732.t1	NP_001137908.1	down syndrome cell adhesion molecule 4, isoform D	3.08E-16	6294473	6308283
g1732.t1	NP_001137908.1	down syndrome cell adhesion molecule 4, isoform D	7.05E-15	6294473	6308283
g1732.t1	NP_001036596.2	down syndrome cell adhesion molecule 4, isoform E	7.04E-35	6294473	6308283
g1732.t1	NP_001036596.2	down syndrome cell adhesion molecule 4, isoform E	6.61E-31	6294473	6308283
g1732.t1	NP_001036596.2	down syndrome cell adhesion molecule 4, isoform E	3.08E-16	6294473	6308283

g1732.t1	NP_001036596.2	down syndrome cell adhesion molecule 4, isoform E	7.05E-15	6294473	6308283
g1732.t1	NP_001036506.1	down syndrome cell adhesion molecule 1, isoform BE	1.95E-30	6294473	6308283
g1732.t1	NP_001036506.1	down syndrome cell adhesion molecule 1, isoform BE	9.84E-29	6294473	6308283
g1732.t1	NP_001036506.1	down syndrome cell adhesion molecule 1, isoform BE	1.69E-19	6294473	6308283
g1732.t1	NP_001036506.1	down syndrome cell adhesion molecule 1, isoform BE	1.26E-15	6294473	6308283
g1732.t1	NP_649078.1	uncharacterized protein, isoform A	3.9E-26	6294473	6308283
g1732.t1	NP_001262027.1	uncharacterized protein, isoform B	3.9E-26	6294473	6308283
g1733.t1	NP_001036506.1	down syndrome cell adhesion molecule 1, isoform BE	3.8E-42	6310968	6333469
g1733.t1	NP_001036506.1	down syndrome cell adhesion molecule 1, isoform BE	1.75E-22	6310968	6333469
g1733.t1	NP_001036506.1	down syndrome cell adhesion molecule 1, isoform BE	6.91E-20	6310968	6333469
g1733.t1	NP_001036506.1	down syndrome cell adhesion molecule 1, isoform BE	1.56E-19	6310968	6333469
g1733.t1	NP_001137909.1	down syndrome cell adhesion molecule 4, isoform H	1.15E-36	6310968	6333469
g1733.t1	NP_001137909.1	down syndrome cell adhesion molecule 4, isoform H	1.75E-23	6310968	6333469
g1733.t1	NP_001137909.1	down syndrome cell adhesion molecule 4, isoform H	1.84E-21	6310968	6333469

g1733.t1	NP_001137908.1	down syndrome cell adhesion molecule 4, isoform D	1.63E-36	6310968	6333469
g1733.t1	NP_001137908.1	down syndrome cell adhesion molecule 4, isoform D	1.84E-23	6310968	6333469
g1733.t1	NP_001137908.1	down syndrome cell adhesion molecule 4, isoform D	2.88E-21	6310968	6333469
g1733.t1	NP_001036596.2	down syndrome cell adhesion molecule 4, isoform E	1.63E-36	6310968	6333469
g1733.t1	NP_001036596.2	down syndrome cell adhesion molecule 4, isoform E	1.84E-23	6310968	6333469
g1733.t1	NP_001036596.2	down syndrome cell adhesion molecule 4, isoform E	2.88E-21	6310968	6333469
g1733.t1	NP_649078.1	uncharacterized protein, isoform A	2.05E-23	6310968	6333469
g1733.t1	NP_001262027.1	uncharacterized protein, isoform B	2.05E-23	6310968	6333469
g1735.t1	NP_001137908.1	down syndrome cell adhesion molecule 4, isoform D	5.34E-33	6351841	6361517
g1735.t1	NP_001137908.1	down syndrome cell adhesion molecule 4, isoform D	4.59E-21	6351841	6361517
g1735.t1	NP_001137908.1	down syndrome cell adhesion molecule 4, isoform D	3.73E-14	6351841	6361517
g1735.t1	NP_001036596.2	down syndrome cell adhesion molecule 4, isoform E	5.34E-33	6351841	6361517
g1735.t1	NP_001036596.2	down syndrome cell adhesion molecule 4, isoform E	4.59E-21	6351841	6361517
g1735.t1	NP_001036596.2	down syndrome cell adhesion molecule 4, isoform E	3.73E-14	6351841	6361517

g1735.t1	NP_001137909.1	down syndrome cell adhesion molecule 4, isoform H	5.48E-33	6351841	6361517
g1735.t1	NP_001137909.1	down syndrome cell adhesion molecule 4, isoform H	4.64E-21	6351841	6361517
g1735.t1	NP_001137909.1	down syndrome cell adhesion molecule 4, isoform H	4.01E-14	6351841	6361517
g1735.t1	NP_001036506.1	down syndrome cell adhesion molecule 1, isoform BE	9.74E-27	6351841	6361517
g1735.t1	NP_001036506.1	down syndrome cell adhesion molecule 1, isoform BE	4.86E-17	6351841	6361517
g1735.t1	NP_001036506.1	down syndrome cell adhesion molecule 1, isoform BE	5.77E-16	6351841	6361517
g1736.t1	NP_649810.1	pink, isoform A	3.19E-63	6363126	6372758
g1736.t1	NP_001303456.1	pink, isoform C	3.19E-63	6363126	6372758
g1736.t1	NP_001303455.1	pink, isoform B	3.19E-63	6363126	6372758
g1737.t1	NP_726612.1	Sox102F, isoform A	1.43E-69	6378212	6488360
g1737.t1	NP_001259074.1	Sox102F, isoform D	1.43E-69	6378212	6488360
g1737.t1	NP_001259073.1	Sox102F, isoform C	1.43E-69	6378212	6488360
g1743.t1	NP_001163715.2	uncharacterized protein, isoform C	1.4E-75	6514980	7122953
g1743.t1	NP_651143.2	uncharacterized protein, isoform B	3.45E-71	6514980	7122953
g1743.t1	NP_001287484.1	uncharacterized protein, isoform D	3.45E-71	6514980	7122953
g1743.t1	NP_001287483.1	uncharacterized protein, isoform C	3.45E-71	6514980	7122953
g1741.t1	NP_001259889.1	earmuff, isoform B	6.19E-40	6918165	6931190

g1741.t1	NP_001259889.1	earmuff, isoform B	9.93E-19	6918165	6931190
g1742.t1	NP_651143.2	uncharacterized protein, isoform B	2.06E-82	6995476	7053560
g1742.t1	NP_001287484.1	uncharacterized protein, isoform D	2.06E-82	6995476	7053560
g1742.t1	NP_001287483.1	uncharacterized protein, isoform C	2.06E-82	6995476	7053560
g1745.t1	NP_524638.3	twin of eyeless, isoform A	8.07E-167	7135073	7189742
g1745.t1	NP_524638.3	twin of eyeless, isoform A	1.11E-28	7135073	7189742
g1745.t1	NP_524628.2	eyeless, isoform A	2.58E-70	7135073	7189742
g1745.t1	NP_524628.2	eyeless, isoform A	4.95E-43	7135073	7189742
g1751.t1	NP_732660.1	torso-like, isoform B	2.52E-123	7256921	7344844
g1751.t1	NP_524440.2	torso-like, isoform A	2.52E-123	7256921	7344844
g1751.t1	NP_001262810.1	torso-like, isoform C	2.52E-123	7256921	7344844
g1753.t1	NP_732660.1	torso-like, isoform B	4.77E-55	7362526	7369847
g1753.t1	NP_524440.2	torso-like, isoform A	4.77E-55	7362526	7369847
g1753.t1	NP_001262810.1	torso-like, isoform C	4.77E-55	7362526	7369847
g1758.t1	NP_524225.1	ripped pocket, isoform A	5.35E-57	7390792	7398754
g1762.t1	NP_524628.2	eyeless, isoform A	3.06E-69	7454928	7460431
g1762.t1	NP_524638.3	twin of eyeless, isoform A	3.3E-65	7454928	7460431

Supplementary Table 5. BLAST results of genes around the region around the associated locus with forewing phenotype against *Heliconius melpomene v2.5*

qseqid	sseqid	pident	evalue	start	end
g1717	HMEL032184g1	95.672	0	6001632	6069730
g1719	HMEL012581g1	72.099	0	6073069	6074392
g1720	HMEL003053g1	87.963	5.04E-148	6082880	6085879
g1721	HMEL003054g1	92.26	0	6086308	6100583
g1722	HMEL003055g1	75.676	6.74E-36	6100590	6104698
g1723	HMEL003056g3	78.543	0	6107821	6110369
g1723	HMEL003056g2	73.711	0	6107821	6110369
g1723	HMEL003056g1.t2	54.251	0	6107821	6110369
g1723	HMEL003056g1	65.405	0	6107821	6110369
g1724	HMEL031494g1	30.182	3.24E-34	6114466	6115794
g1725	HMEL036165g1	39.234	1.46E-38	6115855	6116574
g1726	HMEL034362g1	31.751	3.66E-77	6116731	6118584
g1727	HMEL003057g1	56.294	9.59E-106	6119757	6124412
g1728	HMEL003057g2	62.5	2.12E-102	6125223	6127267
g1729	HMEL003058-RA	93.384	0	6128617	6145667
g1730	HMEL007856-RA	80.074	0	6158862	6200842
g1731	HMEL022357g1	71.288	0	6190393	6207540

g1731	HMEL022357g1	67.955	0	6190393	6207540
g1731	HMEL022357g1	71.563	0	6190393	6207540
g1731	HMEL022357g1	68.632	0	6190393	6207540
g1731	HMEL022357g1	60.864	0	6190393	6207540
g1731	HMEL022357g1	63.368	0	6190393	6207540
g1732	HMEL032188g1	47.758	0	6294473	6308283
g1733	HMEL032188g1	38.809	0	6310968	6333469
g1734	HMEL032188g1	37.571	8.9E-68	6343149	6347605
g1735	HMEL032189g1	53.026	0	6351841	6361517
g1736	HMEL016538g2	73.567	0	6363126	6372758
g1737	HMEL020265g1	91.374	0	6378212	6488360
g1743	HMEL032196g1	74.827	0	6514980	7122953
g1740	HMEL016757g1	29.257	1.35E-50	6907246	6912130
g1741	HMEL011763g1	65.421	0	6918165	6931190
g1742	HMEL011806g1.t2	94.333	0	6995476	7053560
g1742	HMEL011806g1	93.333	0	6995476	7053560
g1744	HMEL012216g1	73.496	0	7121438	7133011
g1745	HMEL012214g1	96.928	0	7135073	7189742
g1746	HMEL032198g1	51.485	4.6E-28	7249146	7250042
g1747	HMEL005533g1	61.538	2.63E-19	7251519	7251945

g1748	HMEL005532g1	29.921	3.97E-21	7253053	7256112
g1751	HMEL032199g1	88.75	0	7256921	7344844
g1751	HMEL032199g1.t2	77.808	0	7256921	7344844
g1749	HMEL005007g1	63.087	3.14E-159	7269375	7274356
g1753	HMEL032200g1	81.953	0	7362526	7369847
g1754	HMEL032201g1	78.761	0	7370488	7382484
g1755	HMEL032202g1	56.25	2.16E-70	7382770	7384085
g1756	HMEL032203g1	65.726	1.54E-126	7384808	7387175
g1757	HMEL032204g1	59.112	0	7387805	7390605
g1758	HMEL032205g1	72.213	0	7390792	7398754
g1759	HMEL009698g1	72.848	3.05E-79	7402208	7409244
g1761	HMEL032207g1	69.565	3.7E-13	7448877	7449965
g1762	HMEL021801g1	88.272	7.78E-101	7454928	7460431

Supplementary Table 6. Transposable elements at the locus associated with forewing pattern identified by RepeatMasker.

Chr.	Start	End	Family	Matching repeat	Position
29	6729052	6729213	RC/Helitron	rnd-4_family-1108	-
29	6730618	6730842	RC/Helitron	rnd-5_family-121	-
29	6730827	6730869	RC/Helitron	rnd-6_family-235	-
29	6731047	6731220	Unknown	rnd-4_family-146	Peak 1
29	6731227	6731396	Unknown	rnd-4_family-279	Peak 1
29	6732514	6732589	RC/Helitron	rnd-6_family-52	Peak 1
29	6733032	6733181	RC/Helitron	rnd-6_family-681	Peak 1
29	6733276	6733314	Simple_repeat	(TATT)n	Peak 1
29	6733396	6733458	LINE/L2	rnd-6_family-627	Peak 1
29	6733459	6734488	RC/Helitron	rnd-4_family-122	Insertion A
29	6734490	6735220	RC/Helitron	rnd-4_family-122	Insertion A
29	6735155	6735671	Unknown	rnd-4_family-53	Insertion A
29	6735672	6735711	Simple_repeat	(GACA)n	Peak 2
29	6735712	6735757	Unknown	rnd-6_family-258	Peak 2
29	6735753	6735835	LINE/L2	rnd-6_family-139	Peak 2
29	6735762	6735870	LINE/L2	rnd-4_family-271	Peak 2
29	6736485	6736644	LINE/R1	rnd-5_family-302	Peak 2

29	6736763	6736792	Simple_repeat	(AT)n	Peak 2
29	6736903	6737006	RC/Helitron	rnd-6_family-235	Insertion B
29	6736923	6737070	RC/Helitron	rnd-4_family-489	Insertion B
29	6736926	6737107	Unknown	rnd-4_family-219	Insertion B
29	6737135	6737534	Unknown	rnd-5_family-759	Insertion B
29	6737537	6738061	LINE/CR1	rnd-6_family-305	Insertion B
29	6738127	6738890	LINE/CR1	CR1-4_Hmel_A	Insertion B
29	6738890	6739670	LINE/CR1	rnd-6_family-79	Insertion B
29	6739680	6740646	LINE/CR1	rnd-6_family-79	Insertion B
29	6740805	6740954	RC/Helitron	rnd-4_family-166	Insertion B
29	6741294	6741310	Unknown	rnd-5_family-927	Peak 3
29	6741311	6741424	Unknown	rnd-5_family-442	Peak 3
29	6741847	6741879	Simple_repeat	(AATAA)n	Peak 3
29	6742085	6742124	Simple_repeat	(ATA)n	Peak 3
29	6742596	6742667	Simple_repeat	(AT)n	Peak 3
29	6742789	6742968	RC/Helitron	rnd-4_family-489	Peak 3
29	6743031	6743064	Unknown	rnd-6_family-306	Peak 3
29	6743065	6743213	RC/Helitron	rnd-4_family-183	Peak 3
29	6743302	6743327	Simple_repeat	(TAA)n	Peak 3
29	6743615	6743692	RC/Helitron	rnd-3_family-741	-

29	6743763	6743827	RC/Helitron	rnd-5_family-201	-
29	6744341	6744593	RC/Helitron	rnd-4_family-148	-
29	6744779	6745035	RC/Helitron	rnd-4_family-149	-
29	6745354	6745470	RC/Helitron	rnd-5_family-178	-
29	6745905	6746033	RC/Helitron	rnd-5_family-897	-
29	6746111	6746336	RC/Helitron	rnd-3_family-741	-
29	6746825	6747024	DNA/TcMar-Mariner	rnd-2_family-183	-
29	6747111	6747156	Simple_repeat	(AT)n	-
29	6747210	6747247	Simple_repeat	(AATA)n	-
29	6747468	6747524	Unknown	rnd-6_family-278	-
29	6747949	6748103	RC/Helitron	rnd-4_family-183	-
29	6748747	6748803	Unknown	rnd-6_family-105	-
29	6748927	6749058	Unknown	rnd-6_family-515	-
29	6749787	6749927	RC/Helitron	rnd-6_family-691	-
29	6749812	6749930	RC/Helitron	rnd-4_family-489	-
29	6749843	6749941	Unknown	rnd-6_family-352	-

References

Lewis JJ, Geltman RC, Pollak PC, Rondem KE, Van Belleghem SM, Hubisz MJ, Munn PR, Zhang L, Benson C, Mazo-Vargas A, et al. 2019. Parallel evolution of ancient, pleiotropic enhancers underlies butterfly wing pattern mimicry. *Proceedings of the National Academy of Sciences* 116:24174–24183.
